# New Insights on Sperm Function in Male Infertility of Unknown Origin: A Multimodal Approach

**DOI:** 10.3390/biom13101462

**Published:** 2023-09-27

**Authors:** Rita I. Pacheco, Maria I. Cristo, Sandra I. Anjo, Andreia F. Silva, Maria Inês Sousa, Renata S. Tavares, Ana Paula Sousa, Teresa Almeida Santos, Mariana Moura-Ramos, Francisco Caramelo, Bruno Manadas, João Ramalho-Santos, Sandra Gomes Amaral

**Affiliations:** 1 CNC—Center for Neuroscience and Cell Biology, University of Coimbra, 3004-504 Coimbra, Portugal; 2 IIIUC—Institute for Interdisciplinary Research, University of Coimbra, 3030-789 Coimbra, Portugal; 3 Department of Life Sciences, Faculty of Sciences and Technology, University of Coimbra, 3000-456 Coimbra, Portugal; 4 Reproductive Medicine Unit, Centro Hospitalar e Universitário de Coimbra, 3000-075 Coimbra, Portugal; 5 Eugin Coimbra, Rua Filipe Hodart 12, 3000-185 Coimbra, Portugal; 6 Faculty of Medicine, University of Coimbra, 3000-548 Coimbra, Portugal; 7 Center for Research in Neuropsychology and Cognitive and Behavioral Intervention, Faculty of Psychology and Educational Sciences, University of Coimbra, 3000-115 Coimbra, Portugal; 8 Clinical Psychology Unit, Centro Hospitalar e Universitário de Coimbra, 3000-075 Coimbra, Portugal

**Keywords:** idiopathic male infertility, unexplained male infertility, sperm function, sperm proteomics, biomarker

## Abstract

The global trend of rising (male) infertility is concerning, and the unidentifiable causes in half of the cases, the so-called unknown origin male infertility (UOMI), demands a better understanding and assessment of both external/internal factors and mechanisms potentially involved. In this work, it was our aim to obtain new insight on UOMI, specifically on idiopathic (ID) and Unexplained male infertility (UMI), relying on a detailed evaluation of the male gamete, including functional, metabolic and proteomic aspects. For this purpose, 1114 semen samples, from males in couples seeking infertility treatment, were collected at the Reproductive Medicine Unit from the Centro Hospitalar e Universitário de Coimbra (CHUC), from July 2018–July 2022. Based on the couples’ clinical data, seminal/hormonal analysis, and strict eligibility criteria, samples were categorized in 3 groups, control (CTRL), ID and UMI. Lifestyle factors and anxiety/depression symptoms were assessed via survey. Sperm samples were evaluated functionally, mitochondrially and using proteomics. The results of Assisted Reproduction Techniques were assessed whenever available. According to our results, ID patients presented the worst sperm functional profile, while UMI patients were similar to controls. The proteomic analysis revealed 145 differentially expressed proteins, 8 of which were specifically altered in ID and UMI samples. Acrosin (ACRO) and sperm acrosome membrane-associated protein 4 (SACA4) were downregulated in ID patients while laminin subunit beta-2 (LAMB2), mannose 6-phosphate isomerase (MPI), ATP-dependent 6-phosphofructokinase liver type (PFKAL), STAR domain-containing protein 10 (STA10), serotransferrin (TRFE) and exportin-2 (XPO2) were downregulated in UMI patients. Using random forest analysis, SACA4 and LAMB2 were identified as the sperm proteins with a higher chance of distinguishing ID and UMI patients, and their function and expression variation were in accordance with the functional results. No alterations were observed in terms of lifestyle and psychological factors among the 3 groups. These findings obtained in an experimental setting based on 3 well-defined groups of subjects, might help to validate new biomarkers for unknown origin male infertility (ID and UMI) that, in the future, can be used to improve diagnostics and treatments.

## 1. Introduction

Currently affecting about 1 in 6 couples worldwide, infertility is a disease with an alarming increasing trend [1] that, for many couples, is more than the non-realization of essential human rights, namely, to decide if, when and how many children they want to father. In fact, this disease has a great social impact, not to mention the psychological distress withstood, which can further contribute to the couple’s inability to conceive, and the heavy financial burden that it might imply [2,3,4].

Male factor alone can contribute to up 20–30% of the cases of infertility, that when associated with female factors, rises up to 50% [5]. The main known causes for male infertility are urogenital and genetic anomalies, genital tract infections, endocrine disorders and immunological factors [6,7,8]. However, some patients present unknown origin male infertility (UOMI) [9,10]. This categorization can be further divided into idiopathic (ID), and unexplained male infertility (UMI), which essentially differ in the seminal analysis results, abnormal in the former and normal in the later, assuming that in both the female factor has been ruled out [11]. ID affects approximately 30 to 40% of infertile men [8], while UMI affects 6 to 30% of infertile men [8,12,13]. In the literature, it is frequent to find different terms to classify these patients, which, together with the frequent lack of proper control groups and the fact that female factor is often disregarded, severely compromise the interpretation of the available information [10,14]. Moreover, considering that the routine seminal analysis is the pillar for male infertility diagnosis, although its limitations in terms of predicting fertility are well-known, additional or improved evaluation tools are needed to understand sperm functionality in full as is a standard and more systematic assessment of potential risk factors, especially for UOMI patients. This stresses the need to deepen the study of the male gametes, ideally focusing on more relevant functional aspects, not routinely evaluated, but that might add knowledge on the mechanisms behind male infertility, hopefully providing the scaffold for the development of new diagnostic tools and treatment options.

Proteomics has a great potential to show changes in protein levels which might explain the mechanism behind male infertility [15], and help define new diagnosis biomarkers. With this approach, Moscatelli and colleagues have previously identified 86 differentially expressed proteins in asthenozoospermic samples compared to normozoospermic, with the most represented proteins being located in the mitochondria, and having a role on metabolism and energy production [16]. Yet, this study did not specifically clarify if the asthenozoospermic samples were from idiopathic infertile patients. A different study, found 16 differentially expressed proteins in idiopathic asthenozoospermic patients, compared to normozoospermic men [17], including proteins associated with sperm motility and involved in management of oxidative stress [17].

Comparing UMI patients to healthy fertile donors, Xu et al. identified 24 differentially expressed proteins, while Panner Selvan et al. have identified 162. Altogether, the observed proteomic alterations on UMI patients led the authors to suggest likely consequences in terms of sperm motility, capacitation, acrosome integrity, cell oxidative damage and survival as well as on gamete communication [18,19,20].

Overall, although some proteomic analyses have been performed focusing specifically on unknown origin male infertility (UOMI) patients, it is relevant to highlight that not only has none specifically focused on ID and UMI together, but also that the proteomic approaches used were different, as were the number of patients involved, the sample processing methods or even the WHO reference guidelines used, warranting further studies on this topic.

In the present study, we performed a complete and integrated analysis of sperm function in well-characterized and defined groups of patients, combined with proteome profiling, aiming to obtain new insights into the potential mechanisms behind UOMI and ideally suggest likely biomarkers for these infertile patients. In the future this knowledge can be used in a clinical setting to improve diagnostic methods, distinguish patients with different types of infertility or improve treatment approaches and decisions.

## 2. Materials and Methods

### 2.1. Chemicals

All chemicals and reagents were acquired from Merck (St. Louis, MO, USA), except the ones specified otherwise.

### 2.2. Individuals’ Recruitment, Diagnostic Work up and Categorization

**Figure 1:**
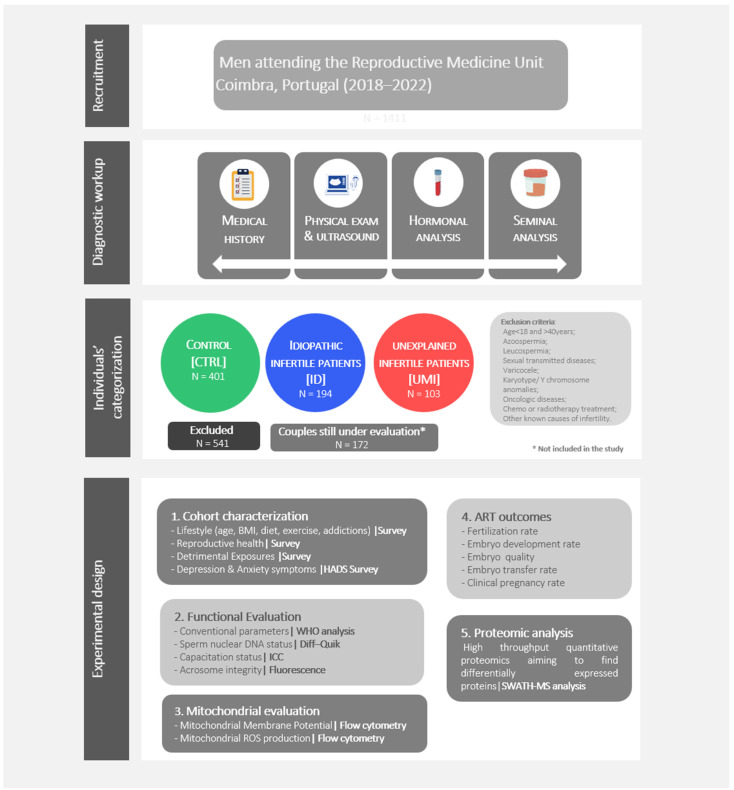
**Study design.** Individuals’ recruitment, diagnostic workup, individuals’ categorization, and experimental design.

Individuals, undergoing routine seminal analysis for fertility treatments, were recruited at the Reproductive Medicine Unit of the Centro Hospitalar e Universitário de Coimbra (CHUC; Coimbra, Portugal) from July 2018 to July 2022. All the individuals in this study have accepted to participate by signing an informed consent form. The medical history, physical examination, hormonal and seminal analysis were part of the diagnostic workup (Figure 1). All procedures were approved by the ethics committee of the CHUC (CHUC-098-18).

Regarding the eligibility criteria, male participants having azoospermia, leucospermia, altered hormone profile, sexual transmitted diseases, varicocele, karyotype/Y chromosome anomalies, oncologic diseases, undergoing or having undergone chemo or radiotherapy treatment or other known causes of infertility were excluded (Figure 1).

On the female side the following criteria were considered for patient exclusion: urogenital anomalies; abnormal gynecological examination and hormonal profile, fallopian tubes altered permeability, oncological disease or associated treatments, urogenital infections and genetic anomalies, being that the absence of these factors exclude the existence of a female factor. The samples categorization in the different fertility/experimental groups was done based on the seminal analysis and the couple’s clinical history, analyzed together with the clinicians, and following the mentioned eligibility criteria.

Therefore, normozoospermic subjects belonging to a couple in which a female factor has been clearly identified were allocated to the Control group (CTRL) while, when in the same circumstances, but without a female factor identified, they were allocated to Unexplained male infertility (UMI) group. Finally, individuals in which the seminal quality is altered in at least one of the evaluated parameters (concentration, motility and morphology), and in which no female factor has been identified, were allocated to Idiopathic infertile men (ID) group.

It is important to mention that the categorization in the 3 mentioned groups (CTRL, ID and UMI), was done following the usual diagnostic workout [21]. In this way, lifestyle/risk factors identified afterwards by survey, were not considered to this diagnostic, but were instead used to ascertain if they might have a role on the fertility status. Additionally, in the control group, 102 individuals have proven fertility and 299 don’t. We have statistically compared these two subgroups and found no differences for all the variables, reason why we didn’t subdivide this group.

Furthermore, all the analyses were done blindly.

#### Semen Samples Collection and Processing

Human semen samples were obtained by masturbation after 3–5 days of sexual abstinence and the seminal analysis was performed in accordance to the World Health Organization (WHO) guidelines [22].

After collection and liquefaction, spermatozoa were separated from other semen constituents by density-gradient centrifugation with SupraSperm (Origio—Cooper Surgical; Ballerup, Denmark) using a 40% (*v*/*v*) density on the top layer and 80% (*v*/*v*) density in the lower layer (WHO, 2010). Briefly, samples were placed on top of the gradient and centrifuged at 400–500× *g* for 10 min. The sperm population recovered from the high-density layer was then resuspended in sperm preparation medium (Origio—Cooper Surgical; Ballerup, Denmark) and allowed to capacitate for at least 3 h at 37 °C and 5% CO_2_ [23].

### 2.3. Cohort Characterization: Socio-Demographic Data, Lifestyle, Reproductive Health, Exposures to Detrimental Agents and Psychological Health

Besides the general characterization (age, BMI, physical exercise, diet, addictions) of the 3 study groups, reproductive health aspects, potential exposure to detrimental agents (at the scope of professional activity) as well as anxiety and depression symptoms were evaluated, in order to have the more accurate and complete characterization possible of the study groups. These aspects were assessed through proper surveys done to all patients enrolled in this study (Figure 1).

The levels of anxiety and depression were assessed through a self-response questionnaire: “Hospital Anxiety and Depression scale” (HADS, Portuguese Version) [24,25]. The HADS is a fourteen-item scale that generates seven items related to anxiety and seven related to depression. A total score of 7 or less is considered “normal”, a score between 8 and 10 is considered “mild”, a score between 11 and 14 is considered a “moderate”, and between 15 and 21 “severe” [26,27].

#### 2.3.1. Sperm Function Evaluation

For the present study, samples were prepared with an adjusted concentration of 10 × 10^6^ spermatozoa/mL in phosphate-buffered saline (PBS) supplemented with 0.9 mM CaCl_2_; 0.5 mM MgCl_2_; 5 mM D-glucose; 25 mM NaHCO_3_; 0.3% (*w*/*v*) BSA; 1 mM sodium pyruvate; 10 mM sodium lactate and 1% (*v*/*v*) penicillin/streptomycin, pH ≈ 7.4 [23], and all the further assays were performed from this stock (Figure 1).

##### Motility and Viability

Both motility and viability were assessed in a phase-contrast optical microscope (Leica—DM4000B; Wetzlar, Germany) at 400× magnification. The eosin exclusion assay was used to assess viability [22]. For each condition a total of 100 spermatozoa were evaluated in different fields. The results were expressed as the percentage of motile spermatozoa (progressive plus non-progressive motility) and percentage of viable spermatozoa, respectively.

##### Sperm Morphology and Chromatin Status

Morphology and sperm chromatin status were assessed by bright field optical microscopy (Leica DM4000B; Wetzlar, Germany), at 1000× magnification, both through the Diff-Quik staining (Millipore—#1.11674.0001; Burlington, MA, EUA), as previously described [28]. For both parameters a total of 100 spermatozoa were evaluated in different fields, and the results were expressed as the percentage of normal spermatozoa [22] and percentage of spermatozoa with higher chromatin integrity [28], respectively.

##### Capacitation Status: Assessment of Tyrosine Phosphorylation

The capacitation status of spermatozoa was evaluated by the detection of phosphotyrosine residues by immunocytochemistry (ICC), as previously described [29]. Briefly, spermatozoa were incubated with a rabbit anti-phosphotyrosine polyclonal antibody (1:10; ThermoFisher—#61-5800; Waltham, MA, USA), overnight at 37 °C, and after labelling with the secondary fluorescent antibody [23] for 1 h at 37 °C, mounted on glass slides with Vectashield Mounting Media containing 4′, 6-diamidino-2-phenylindole (DAPI) (Vector Labs—#H1200; Newark, CA, USA), that counterstain the DNA.

Capacitation status was then assessed using a Leica fluorescence microscope (Leica—DM4000B; Wetzlar, Germany), at 1000× magnification and for each sample, a total of 100 spermatozoa were evaluated in different fields. Spermatozoa were then categorized as follows: (a) stained tail with bright fluorescence; (b) partially stained tail; (c) dot-stained tail and (d) very weak or non-existent fluorescence. The first three categories indicate capacitated spermatozoa while the last entails non-capacitated spermatozoa. The results were presented as the percentage of capacitated sperm [23].

##### Acrosomal Integrity

Acrosome integrity was evaluated by fluorescence microscopy (Leica—DM4000B; Wetzlar, Germany) using a marker for the acrosomal content, the PSA-FITC (*Pisum sativum* agglutinin linked to fluorescein isothiocyanate). Samples were incubated with PSA-FITC (1:200; Sigma—#L0770; Merck; St. Louis, MO, USA) for 1 h at 37 °C and mounted with Vectashield Mounting Media with 4′, 6-diamidino-2-phenylindole (DAPI) as described previously (Vector Labs—#H1200; Newark, CA, USA) [23].

A total of 100 spermatozoa, in different fields at 1000× magnification, were evaluated for acrosomal integrity assessment. Sperm cells were then categorized as follows: (a) cells with bright green homogeneous fluorescence and (b) cells with heterogeneous spots of fluorescence, only a fluorescing band at the equatorial segment or no fluorescence, that indicate intact acrosomes and reacted or about to react acrosomes, respectively. The results were expressed as the percentage of spermatozoa with intact acrosome [23].

#### 2.3.2. Sperm Mitochondrial Functionality Evaluation

Mitochondrial membrane potential (MMP) and sperm mitochondrial superoxide production, were monitored by flow cytometry using two different fluorescent probes: the JC-1 and Mitosox Red, respectively. After incubation with the specific fluorescent probes, sample analysis was performed on a FACSCalibur flow cytometer (BD Biosciences; Franklin Lakes, NJ, USA). This cytometer is equipped with an argon laser that has an excitation wavelength of 488 nm and three emission filters: 530/30 band pass (FL-1/green), 585/42 band pass (FL2/red) and >620 nm long pass filter (FL3/far red). Spermatozoa have specific characteristics of light scatter [forward (FSC) and side scatter (SSC)] and based on that, unspecific events were gated out of the analysis. A total of 15,000 events were recorded per sample, and the data acquisition and analysis were achieved using the BD Cell Quest Pro Acquisition program [23].

##### Sperm Mitochondrial Membrane Potential

Sperm MMP was evaluated using the fluorescent probe 5,5′,6,6′-tetrachloro-1,1′,3,3′-tetraethylbenzimi-dazolycarbocyanine iodide (JC-1^®^; Thermofisher—#T3168; Waltham, MA, USA), a dynamic probe that changes its fluorescence according to the MMP [29,30].

As previously described, sperm cells (2.5 × 10^6^) were incubated with 2 µM of JC-1^®^ during 15 min at 37 °C in the dark and for each sample appropriate controls were prepared: a negative control in which the cells were incubated without the fluorescent probe and a positive control in which cells were incubated simultaneously with JC-1^®^ and 50 µM of carbonyl cyanide 3 chlorophenylhydrazone (CCCP), a mitochondrial uncoupler that disrupts MMP [30,31]. Results were expressed as the percentage of spermatozoa with high MMP.

##### Sperm Mitochondrial Superoxide Production

Sperm mitochondrial superoxide production was assessed using the fluorescent probe MitoSox Red^®^ (Thermofisher—#M36008; Waltham, MA, USA). Sperm cells (2.5 × 10^6^) were incubated with 3 µM of MitoSOX-Red for 15 min at 37 °C in the dark and for each sample suitable controls were prepared: a negative control in which cells were incubated without the fluorescent probe and a positive control in which cells were simultaneously incubated with the fluorescent probe and 80 µM of the complex III inhibitor, antimycin A, known to increase the production of superoxide anion, as previously described [23,30,31]. Results were expressed as fluorescence intensity.

#### 2.3.3. Fertility Outcomes

A total of 123 cycles were included in our analysis, corresponding to patients that performed Assisted Reproductive Technology (ART) treatments and were already diagnosed. Fertility results from IVF and ICSI were evaluated.

Fertility rate (number of oocytes with two pronucleus/number of oocytes that were inseminated or injected), Embryo development rate (number of embryos/number of oocytes with two pronuclei), and Embryo Transfer rate (number of transfers/number of embryos) were determined, and Clinical pregnancy was determined as the number of couples with positive biochemical pregnancy. The results were expressed as mean ± standard error of the mean (SEM) [32].

#### 2.3.4. Sperm Proteomics

For the quantitative proteomic analysis, samples were subjected to Short GeLC digestion and the resulting peptides were analysed by liquid chromatography coupled to tandem mass spectra (LC-MS/MS) using the SWATH-MS acquisition mode [33].

##### Protein Solubilization and Digestion

Briefly, 3 to 5 million/mL spermatozoa were centrifuged (500× *g*, 5 min) followed by resuspension in, respectively 30 to 50 μL of sample buffer [0.25 M Tris-HCl, 4% sodium dodecyl sulfate (SDS), 20% glycerol, 1 mg bromophenol blue, 200 mM dithiothreitol (DTT)] with 10 μg/mL recombinant MBP-GFP [34]. The samples were sonicated at 50% output for 1 min with 5 s bursts at 3 s intervals, using a Hielscher sonifier (Hielsher ultrasonics—UP100H; Teltow, Germany), and boiled for 5 min at 95 °C. Samples were then centrifuged (20,000× *g*, 15 min) and the supernatants were stored at −80 °C [33]. For protein identification/library generation, 3 pooled samples were created: one from control samples, a second one from ID samples, and a third one from UMI samples. Additionally, to test the reproducibility of the method, a pool containing part of all samples studied was created to be used as technical replicate. All the samples were subjected to trypsin digestion using the Short-GeLC approach [33].

##### Protein Identification and Quantification

Samples were analyzed on a NanoLC™ 425 System (Eksigent^®^; Framingham, MA, USA) coupled to a TripleTOF™ 6600 System (Sciex^®^; Framingham, MA, USA) using information-dependent acquisition (IDA) for the pooled samples and SWATH-MS acquisition of each individual sample for protein quantification. Samples were loaded onto a YMC-Triart C18 Capillary Guard Column 1/32” (12 nm, S-3 μm, 5 × 0.5 mm) (YMC; Kyoto, Japan) at 5 μL/min of 5% of mobile phase B during 8 min and the peptides separation was carried out by micro-flow liquid chromatography using a YMC-Triart C18 Capillary Column (12 nm, S-3 μm, 150 × 0.3 mm) at 50 °C. The flow rate was set to 5 μL/min and mobile phases A and B were 5% DMSO plus 0.1% formic acid in water and 5% DMSO plus 0.1% formic acid in acetonitrile, respectively. The LC program was performed as follows: 5–30% of B (0–50 min), 30–98% of B (50–52 min), 98% of B (52–54 min), 98–95% of B (54–56 min), 95% of B (56–65 min). Peptides were eluted into the mass spectrometer using an electrospray ionization source (ABSciex^®^—DuoSpray™ Source; Framingham, MA, USA) with a 25 μm internal diameter hybrid PEEKsil/stainless steel emitter (ABSciex^®^; Framingham, MA, USA). The ionization source was operated in the positive mode set to an ion spray voltage of 5500 V, 25 psi for nebulizer gas 1 (GS1), 10 psi for nebulizer gas 2 (GS2), 25 psi for the curtain gas (CUR), and source temperature (TEM) at 100 °C.

For IDA experiments, the mass spectrometer was set to scanning full spectra (*m*/*z* 350–2250) for 250 ms, followed by up to 100 MS/MS scans (*m*/*z* 100–1500) per cycle, in order to maintain a cycle time of 3.295 s. The accumulation time of each MS/MS scan was adjusted in accordance with the precursor intensity (minimum of 30 ms for precursor above the intensity threshold of 2000). Candidate ions with a charge state between +1 and +5 and counts above a minimum threshold of 10 counts/s were isolated for fragmentation and one MS/MS spectra was collected before adding those ions to the exclusion list for 15 s (mass spectrometer operated by Analyst^®^ TF 1.8.1, Sciex^®^; Framingham, MA, USA). Rolling collision was used with a collision energy spread of 5.

For SWATH-MS experiments, the mass spectrometer was operated in a looped product ion mode [35] with the same chromatographic conditions used in the IDA run described above. A set of 168 windows of variable width (containing an *m*/*z* of 1 for the window overlap) was constructed, covering the precursor mass range of *m*/*z* 350–2250. A 50 ms survey scan (*m*/*z* 350–1250) was acquired at the beginning of each cycle for instrument calibration and SWATH MS/MS spectra were collected from the precursors ranging from *m*/*z* 350 to 1250 for *m*/*z* 100–1500 for 19 ms resulting in a cycle time of 3.291 s The collision energy (CE) applied to each *m*/*z* window was determined considering the appropriate CE for a +2 ion centered upon this window and the collision energy spread was also adapted to each *m*/*z* window.

By combining all files from the IDA experiments, a specific library containing the precursor masses and fragment ions was created and used for subsequent SWATH processing. Libraries were obtained using ProteinPilot™ software (ABSciex^®^ v5.0.1; Framingham, MA, USA), in accordance with the following parameters: (i) search against a database from SwissProt composed by Homo Sapiens (downloaded in August 2021) and MBP-GFP (IS) protein sequences; (ii) acrylamide alkylated cysteines as fixed modification; (iii) trypsin as digestion type. An independent False Discovery Rate (FDR) analysis using the target-decoy approach provided with Protein Pilot software was performed to assess the quality of the identifications. Positive identifications were considered when identified proteins and peptides reached a 5% local FDR [36,37].

Data processing was performed using SWATH™ processing plug-in for PeakView™ (ABSciex^®^; Framingham, MA, USA) v2.0.01 [38]. After retention time adjustment using peptides from the internal standard (MBP-GFP) [39], up to 15 peptides, with up to 5 fragments each, were chosen per protein, and extracted-ion chromatograms (XIC) were attempted for all proteins in library file that were identified from ProteinPilot™ search. Only proteins with at least one confidence peptide (FDR < 0.01) were considered in no less than four replicates condition and with at least three transitions. Peak areas of the target fragment ions (transitions) of the retained peptides were extracted across the experiments using an XIC window of 6 min with 100 ppm XIC width. The proteins’ levels were estimated by summing all the transitions from all the peptides for a given protein that met the criteria described above [34] and normalized to the levels of two spermatozoa-specific proteins (AKAP4 + AKAP3) [39].

The mass spectrometry proteomics data have been deposited to the ProteomeXchange Consortium via the PRIDE partner repository with the dataset identifier [40].

#### 2.3.5. Statistical Analysis

Statistical analysis was performed using the SPSS software (Statistical Package for the Social Sciences Program) version 20.0 for windows (SPSS Inc.; Chicago, IL, USA). All variables were evaluated for normal distribution through Shapiro-Wilk or Kolmorogov-Smirnov test, and the Levene’s test assessed the homogeneity of variances. The comparison between study groups, was assessed using the Kruskal-Wallis test, followed by the Mann-Whitney U test to assess specific differences among groups or the Chi-square (*x*^2^) test, according to the nature of the variables. To assess the correlation between variables, the Spearman coefficient was computed. Linear regressions were performed whenever justified and the odds ratio calculated. Values of *p* < 0.05 were considered statistically significant and the results were expressed as mean ± SEM. The number of experiments was always indicated.

The statistical analysis regarding the proteomic data was done using R, as follows: a Kruskal-Wallis test was performed to identify the proteins differentially regulated between all the comparisons followed by the Dunn’s test of Multiple Comparisons, with Benjamini–Hochberg *p*-value correction, to determine in which comparisons statistical differences were observed. All analyses were performed using the normalized protein levels and a *p*-value of 0.05 was defined as a cut-off.

From all the altered proteins, the ones with only 1 peptide quantified and a coefficient of variation (CV) among technical replicates higher than 20% were excluded. Protein Analysis Through Evolutionary Relationships (PANTHER) was used to analyse the different biological processes present in proteins up- and down-regulated between the patient’s groups (August 2022) [41].

Data Visualization and Integrated Discovery (DAVID) functional annotation for KEGG pathways and gene ontology (GO) term (August 2022), and cluster analysis (October 2022) [42], FunRich transcription factor analysis (August 2022) [43], and MetaboAnalyst 5.0 random forest analysis (September 2022) [44] were also performed to better characterize the altered proteins.

## 3. Results

### 3.1. Samples Categorization in the 3 Study Groups

As previously mentioned, the categorization of the collected human samples in the 3 study groups was based on a comprehensive analysis of the clinical history of the couple, on the seminal analysis, and followed strict eligibility criteria (Figure 1).

We collected 1411 samples, of which 401 were classified as CTRL, 194 as ID and 103 as UMI. Samples that did not fit the inclusion criteria were excluded, corresponding to 541 samples. Additionally, in 172 samples a diagnosis was not possible, as the couples are still being evaluated and for that reason, they were not included in the study (Figure 1).

### 3.2. Cohort Characterization—Lifestyle, Reproductive Health, Occupational Exposures, and Psychological Health

Age, BMI, regular physical exercise, eating habits, chronic diseases and the existence of other diseases were not significantly different between groups (*p* > 0.05; Table S1). On the other hand, ID patients had a higher prevalence of hypercholesterolemia (*p* < 0.05) and were also more frequently submitted to surgeries (*p* < 0.001) than the individuals from the other groups (Table S1). Surprisingly, the ID group had a lower percentage of individuals that had COVID 19 (*p* < 0.05) whereas the CTRL group seemed to suffer more from allergies, when compared to UMI (Table S1).

Most of the reproductive health aspects assessed by survey (including the STDs, urogenital infections and pre-existing varicocele, urogenital anomalies, testicular torsion, inguinal hernia, and hormonal therapy) were not statistically different among the three groups (*p* > 0.05), with the exception of urogenital infections and pre-existing varicocele (CTRL: 7.0%; ID: 22.9%; UMI: 3.9%; *p* < 0.001), the prevalence of which was significantly increased in the ID patients’ group when compared to the control and UMI group. Furthermore, the chance of belonging to the ID group vs. UMI group is seven times higher in individuals with varicocele (Expected-odds ratio 0.137). Similarly, the urogenital anomalies were also observed to be more prevalent in the ID group (CTRL: 0.7%; ID: 3.1%; UMI: 0.0%; *p* < 0.05; Table S2).

Occupational exposure to different potentially detrimental agents for more than 3 months, such as paints, solvents, pesticides, metals, high and/or low temperatures, radiation and dust were not significantly different between the three studied groups (*p* > 0.05), with the exception of paints and metals. In fact, and unexpectedly, the control group seems to be more exposed to paints compared to the study groups (CTRL: 26.4%; ID: 17.0%; UMI: 19.4%; *p* < 0.05) while the group that was less exposed to metals was the ID one (CTRL: 31.5%; ID: 21.4%; UMI: 31.3%; *p* < 0.05; Table S3). Regarding alcohol and tobacco consumption, no statistically significant differences were observed (Table S4).

Concerning the psychological state, anxiety and depression symptoms, evaluated by the HADS questionnaire, were not statistically significantly different among the three study groups (*p* > 0.05). Yet, it seems that when compared to the other groups, the ID patients tend to present higher anxiety scores and the UMI patients higher depression scores (Table S5), despite being in the normal range, according to HADS manual [24]. Nevertheless, UMI patients do present a significantly higher percentage of men with diagnosed depression compared to CTRL and ID patients, as assessed on the survey, and the patients that have/had depression have 4 times higher chance of being in the UMI group than in the CTRL (OR = 0.256; *p* < 0.05; Table S5).

### 3.3. Functional Sperm Parameters

Motility, viability and normal morphology were significantly lower in ID patients when compared to both control and UMI patients (*p* < 0.001; Table 1).

**Figure 2:**
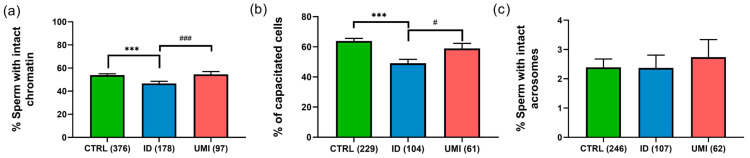
Sperm non-conventional parameters. (**a**) Percentage of cells with intact chromatin; (**b**) Percentage of capacitated cells; (**c**) Percentage of cells with intact acrosome. Data are presented as mean ± SEM and the number of experiments is indicated in brackets; ***—*p* < 0.001 in comparison to CTRL; ^###^—*p* < 0.001 and ^#^—*p* < 0.05 in comparison with ID. CTRL—healthy men, ID—idiopathic infertile men, UMI—unexplained infertile men.

Sperm chromatin integrity was also significantly decreased in ID patients (46.74 ± 1.78) compared to CTRL (53.86 ± 1.23) and UMI patients (54.54 ± 2.47; *p* ≤ 0.05), although no statistically significant differences were observed between CTRL and UMI patients (*p* > 0.05; Figure 2a).

Regarding capacitation, this process seemed to be affected in the ID group, when compared to both the control (*p* < 0.001) and the UMI groups (*p* < 0.05; Figure 2b). On the other hand, the percentage of cells with intact acrosome was similar among all study groups (CTRL: 2.39 ± 0.29; ID: 2.37 ± 0.44; UMI: 2.73 ± 0.60; *p* > 0.05; Figure 2c).

### 3.4. Sperm Mitochondrial Functionality

**Figure 3:**
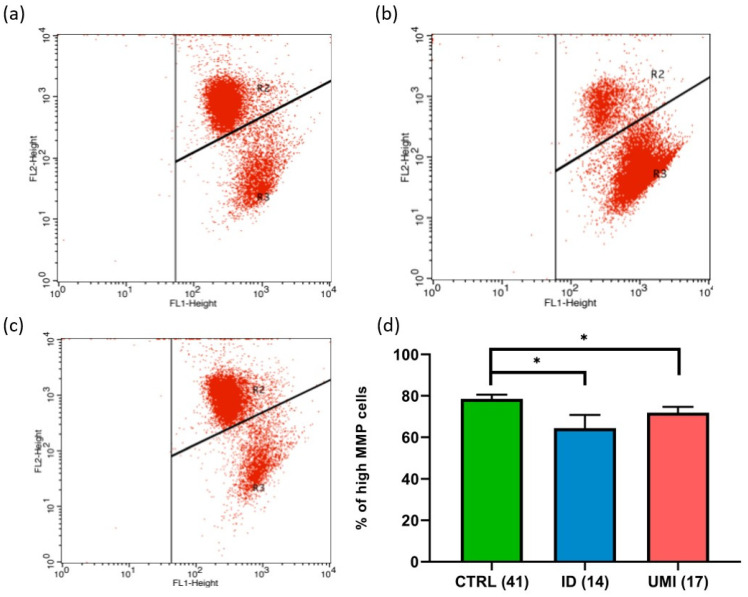
Sperm Mitochondrial membrane potential (MMP). Representative flow cytometry dot-plot chart of (**a**) CTRL, (**b**) ID, and (**c**) UMI sperm cells incubated with JC-1. (**d**) Percentage of cells with high MMP on the three study groups. Data are presented as Mean ± S.E.M. and the number of experiments is indicated in brackets; *—*p* < 0.05 in comparison to CTRL. CTRL—healthy men, ID—idiopathic infertile men, UMI—unexplained infertile men.

**Figure 4:**
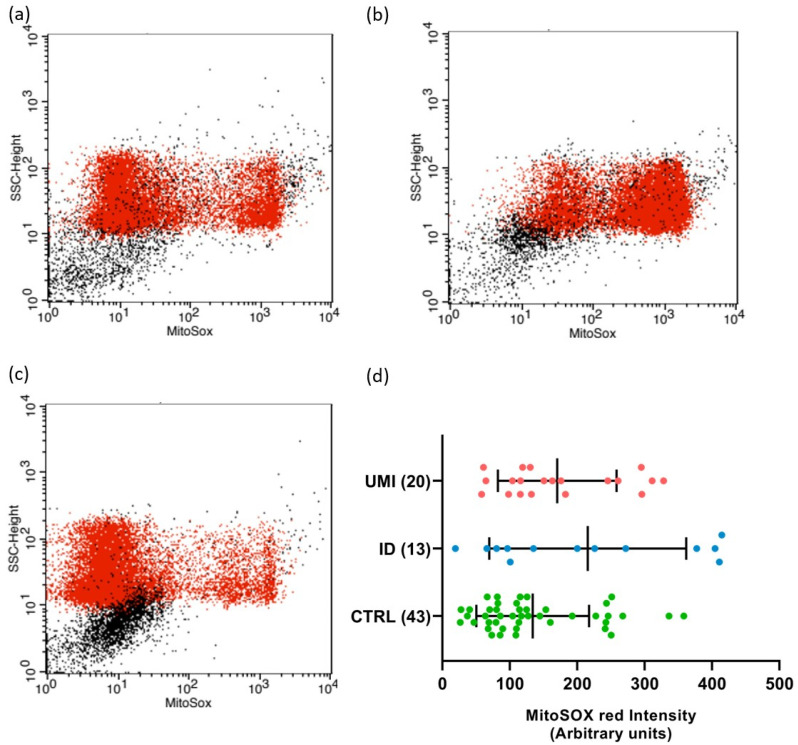
Sperm mitochondrial superoxide levels. Flow cytometry dot-plot chart of CTRL (**a**), ID (**b**), and (**c**) UMI sperm cells labelled with MitoSOX (red dots). (**d**) MitoSOX fluorescence intensity (arbitrary units) on the three study groups. Data are presented as mean ± SEM and the number of experiments is indicated in brackets. CTRL—healthy men, ID—idiopathic infertile men, UMI—unexplained infertile men.

Due to the strong association between sperm and mitochondrial (dys)function [30], the evaluation of mitochondria-related parameters has gained support as a trustworthy and easy readout of sperm quality. Indeed, in this study, the mitochondrial membrane potential was affected in both infertile groups in comparison to the control (*p* < 0.05). Additionally, a trend towards higher ROS levels was also observed in the ID group (*p* = 0.092), although not reaching statistical significance (*p* > 0.05; Figure 3 and Figure 4).

### 3.5. Fertility Outcomes

Fertility outcomes constitute an important measure of the gametes’ quality and fertilization success that can be further related to the other evaluated parameters in an attempt to find the best to predict these outcomes. Nevertheless, no statistically significant differences were detected between the study groups in all calculated rates: fertilization, embryo development, and embryo transfer rates (*p* > 0.05; Table 2).

### 3.6. Correlations

Regarding correlations between functional parameters, as expected, cells with higher MMP were positively correlated with sperm viability (r = 0.620, *p* < 0.01), motility (r = 0.679, *p* < 0.01) and capacitation (r = 0.464, *p* < 0.01), and negatively correlated with cells with higher production of superoxide (r = −0.839, *p* < 0.01; Figure S1).

Interestingly, the presence of urogenital infections and varicocele were negatively correlated with all functional parameters except for morphology (*p* < 0.01), and positively correlated with urogenital anomalies and surgeries (*p* < 0.01; Figure S1). In turn, surgeries were also positively correlated with urogenital infections and varicocele and negatively correlated with some sperm parameters, like, viability, motility, and chromatin status (*p* < 0.05; Figure S1).

Hypercholesterolemia was correlated with all seminal parameters, including mitochondrial ones. Most of these correlations were negative with the exception of the one with cells with higher superoxide production, and, unexpectedly, also with acrosome status (*p* < 0.01; Figure S1).

### 3.7. Sperm Proteomics

**Figure 5:**
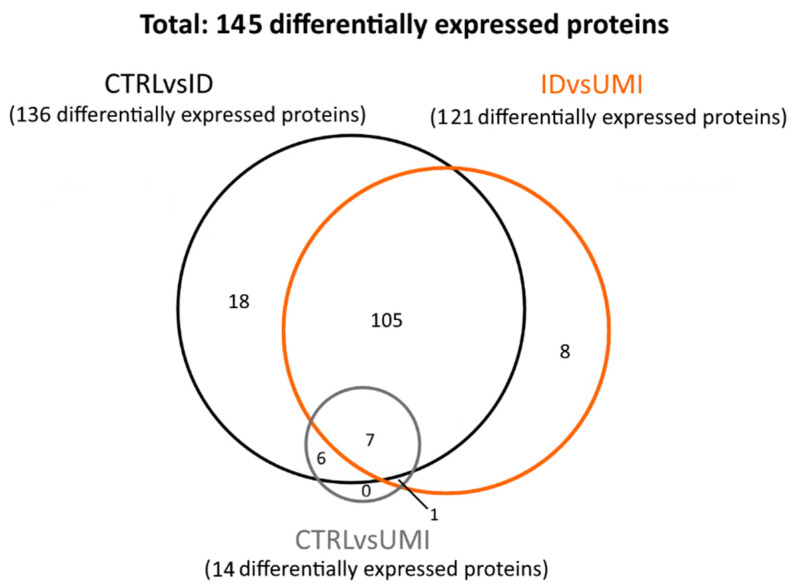
Venn diagram reflecting the number of differentially expressed proteins between the study groups. CTRL—healthy men, ID—idiopathic infertile men, UMI—unexplained infertile men.

The proteomic analysis included 79 samples: 50 CTRL, 19 ID and 10 UMI (Supplementary File S1). In these samples, 295 proteins were found to be differentially expressed (*p* < 0.05). Proteins where only one peptide was detected and the coefficient of variation on the technical replicates was higher than 20% were excluded, leaving 145 proteins for subsequent analysis. These proteins were then distributed in three protein sets: (1) proteins differentially expressed between CTRL and ID (CTRLvsID), containing 136 proteins; (2) proteins differentially expressed between CTRL and UMI (CTRLvsUMI), containing 14 proteins, and proteins differentially expressed between ID and UMI (UMIvsID), containing 121 proteins (Figure 5).

**Figure 6:**
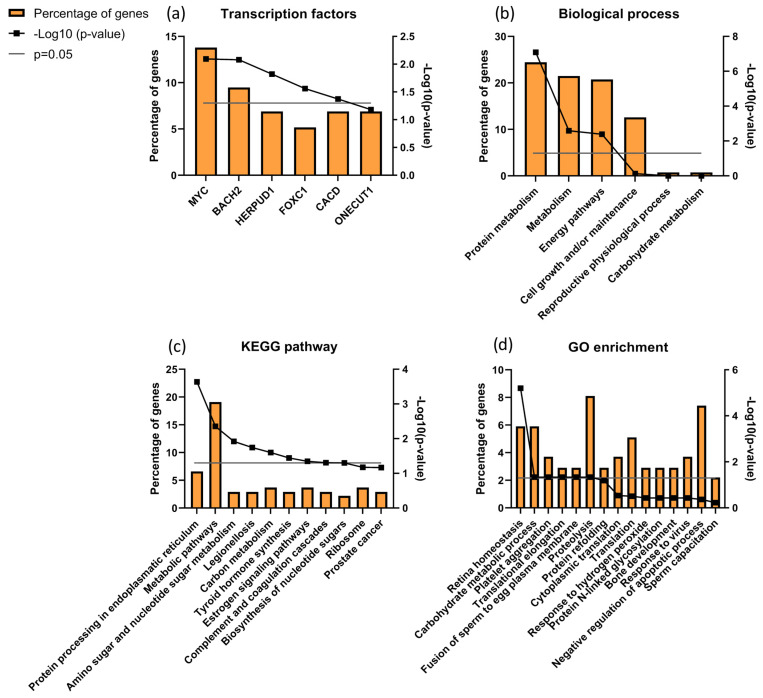
Characterization of the groups of proteins for (**a**) transcription factors; (**b**) biological process; (**c**) KEGG pathways and (**d**) GO enrichments possibly associated with male infertility. Grey line corresponds to *p* value = 0.05. If the black line squares are above the grey line, the gene is considered enriched. In (**d**) the corrected *p* value (Benjamini) was used.

Focusing the analysis on CTRL versus infertile patients (ID and UMI), 137 proteins were identified to be differentially expressed, corresponding to the combination of CTRLvsID with CTRLvsUMI (18 + 105 + 7 + 6 + 1 + 0 = 137; Figure 5). These proteins were characterized based on possible transcription factors involved, biological process, KEGG pathways and GO enrichment (Figure 6). MYC was found to be the most enriched transcription factor involved in this protein group (Figure 6a). On the other hand, the top five biological processes among proteins differentially expressed between CTRL and infertile patients were *protein metabolism*, *metabolism*, *energy pathways*, *cell growth and/or maintenance*, and *physiological reproductive process* although the latter was not significantly enriched (Figure 6b). Furthermore, *protein processing in the endoplasmic reticulum, carbon metabolism*, *thyroid hormone synthesis*, and *estrogen signaling pathways* were some of the KEGG pathways found to be relevant for this protein group (Figure 6c). *Fusion of sperm to the egg plasma membrane*, *response to hydrogen peroxide* and *sperm capacitation* were among the relevant GO terms found in this protein group (Figure 6d).

Through DAVID cluster analysis, we found three relevant clusters of proteins: one composed of Annexins (3.55 enrichment score), a second one composed of protein associated with refolding/stress response (1.56 enrichment score) and another associated with the serpin family (1.38 enrichment score; Figure S2). The first cluster was composed of Annexin 2 (ANXA2), 3 (ANXA3), 5 (ANXA5) and 6 (ANXA6), all significantly upregulated in ID patients compared to CTRL. The second cluster included proteins α-crystallin B chain (CRYAB), Heat shock 70 kDa protein 1B (HS71B), Heat shock cognate 71 kDa protein (HSP7C), Heat shock protein β1 (HSPB1) and endoplasmin related with protein refolding and stress response, all downregulated in CTRL comparing to ID patients. The third cluster was composed of 5 serpin family proteins: plasma serine protease inhibitor (IPSP/SERPINA5), α-1-antitrypsin (A1AT/SERPINA1), antithrombin-III (ANT3/SERPINC1), ANXA2 and clusterin, which were all downregulated in CTRL comparing to ID, except for ANT3 which was upregulated (*p* < 0.05).

**Figure 7:**
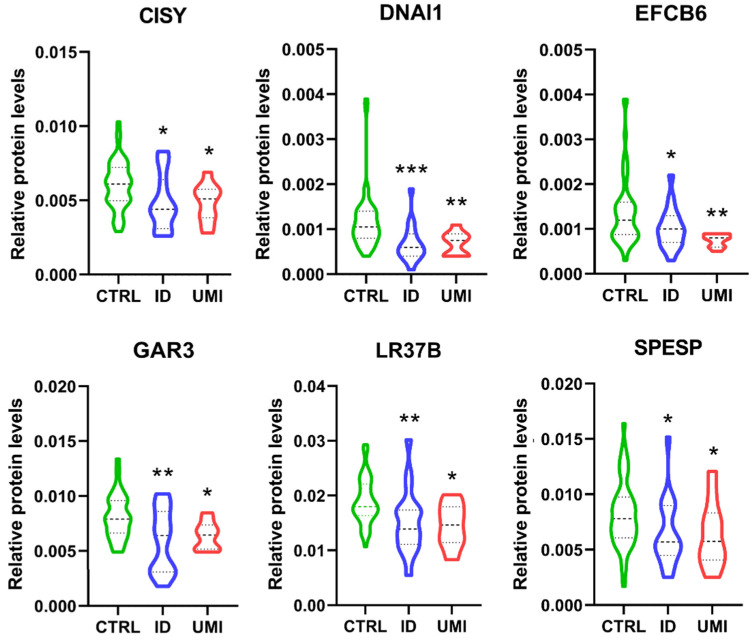
Normalized proteins levels of the proteins differently altered between the fertile group and both types of infertile patients. CISY—citrate synthase. DNAI1—Dynein axonemal intermediate chain 1, EFCB6—EF-hand calcium-binding domain-containing protein 6, GAR3—Golgi-associated RAB2 interactor protein 3, LR37B—Leucine-rich repeat-containing protein 37B, SPESP—Sperm equatorial segment protein 1. *—*p* < 0.05 in comparison to CTRL; **—*p* < 0.01 in comparison to CTRL; ***—*p* < 0.001 in comparison to CTRL. CTRL—Healthy men; ID—idiopathic infertile men; UMI—unexplained infertile men.

Furthermore, only 6 proteins were differentially expressed between CTRL and both infertile groups (ID and UMI). These proteins were mitochondrial citrate synthase (CISY), Dynein axonemal intermediate chain 1 (DNAI1), EF-hand calcium-binding domain-containing protein 6 (EFCB6), Golgi-associated RAB2 interactor protein 3 (GAR3), Leucine-rich repeat-containing protein 37B (LR37B) and Sperm equatorial segment protein 1 (SPESP), with the mean protein’s levels significantly lower in the infertile groups comparing to CTRL (Figure 7).

Finally, the Random Forest analysis found that these 6 proteins, together with functional sperm parameters described previously, can accurately classify patients in fertile or infertile with out-of-bag error of 0.215, where DNAI1 levels was considered as the most relevant factor for this discrimination (Figure S3).

**Figure 8:**
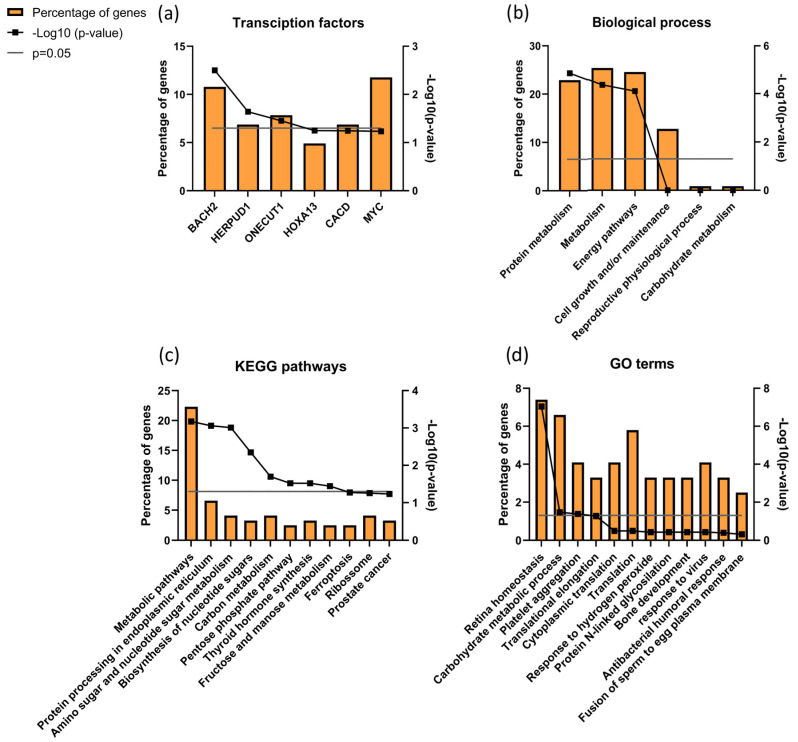
Characterization of the groups of proteins for (**a**) transcription factors, (**b**) biological process, (**c**) KEGG pathways, and (**d**) GO enrichments possibly associated with unknown origin (ID and UMI) male infertility. Grey line corresponds to *p* value = 0.05. If the black line squares are above the grey line, the gene is considered enriched. In (**d**) the corrected p value (Benjamini) was used.

When analyzing proteins differentially expressed between ID and UMI patients, 121 were found corresponding to the IDvsUMI group (105 + 8 + 7 + 1 = 121; Figure 5). This new group of proteins was also characterized based on possible transcription factors involved, biological process, KEGG pathways and GO enrichment (Figure 8). BACH2 was found to be the most enriched transcription factor involved with this protein group (Figure 8a). The top five biological processes among proteins differentially expressed between ID and UMI patients were *protein metabolism*, *metabolism*, *energy pathways*, *cell growth and/or maintenance*, and *reproductive physiological process* (Figure 8b). *Pentose phosphate pathway*, *thyroid hormone synthesis*, and *fructose and mannose metabolism* are enriched KEGG pathways associated with proteins differentially expressed between ID and UMI patients (Figure 8c). On the other hand, *carbohydrate metabolic process*, *response to hydrogen peroxide* and *fusion of sperm to egg plasma membrane* are relevant GO terms associated with these proteins (Figure 8d), that despite not being statistically significant, are worth mentioning given the scope of this study.

Through DAVID cluster analysis, we found glycosidase activity as a relevant cluster (1.38 enrichment score) including β-galactosidase-1-like protein (GLB1L), neutral α-glucosidase AB (GANAB) and maltase-glucoamylase (MGA; Figure S4). GANAB and MGA were significantly upregulated in ID comparing to CTRL, while GLB1L was significantly downregulated in ID patients compared to CTRL. Furthermore, 8 proteins were found differentially expressed exclusively between ID and UMI patients (Table 3).

**Figure 9:**
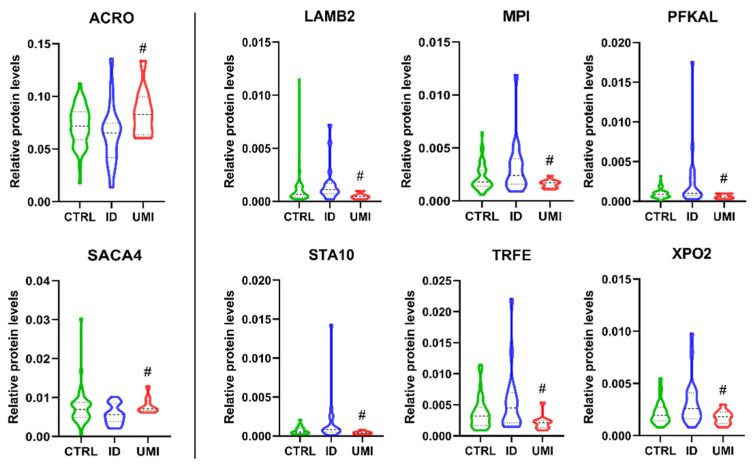
Normalized proteins levels of the proteins differently altered between ID and UMI. ACRO—acrosin, SACA4—sperm acrosome membrane-associated protein 4, LAMB2—laminin subunit beta-2, MPI—mannose-6-phosphate isomerase, PFKAL—ATP-dependent 6-phosphofructokinase liver type, STA10—START domain-containing protein 10, TRFE—serotransferrin, XPO2—exportin-2. #—*p* < 0.05 in comparison to ID; CTRL—Healthy men; ID—idiopathic infertile men; UMI—unexplained infertile men.

Acrosin (ACRO) and sperm acrosome membrane-associated protein 4 (SACA4) were significantly down-regulated in ID patients compared to UMI (*p* < 0.05; Figure 9), while Laminin subunit beta-2 (LAMB2), mannose-6-phosphate isomerase (MPI), ATP-dependent 6-phosphofructokinase liver type (PFKAL), START domain-containing protein 10 (STA10), serotransferrin (TRFE) and exportin-2 (XPO2) were significantly down-regulated in UMI patients compared to ID (*p* < 0.05) (Figure 9).

Random forest analysis found that these 8 proteins together with functional sperm parameters can accurately classify patients into ID or UMI with an out-of-bag error of 0.276, where LAMB2 and SACA4 peak intensities were the most accurate factors (Figure S5).

## 4. Discussion

Male infertility of unknown origin, more specifically idiopathic and unexplained infertility, differs essentially in terms of seminal quality [76,77]. Seminal analysis has not changed much in the past fifty years, and there is now evidence that it cannot accurately predict the fertilization capacity of the spermatozoa from the evaluated men [78]. In fact, apparently fertile men may be unable to have a biological child [79], while a presumable infertile man might end up conceiving [80]. Furthermore, the categorization in ID or UMI is not always straightforward and varies quite a lot among the available studies [81,82]. The contradictions and gaps found in the literature regarding these types of infertility highlight the need to study these patients more deeply, focusing both on clear characterization and on aspects that are not routinely evaluated, a need also identified in a recent publication by Corsini and colleagues [83].

Our comprehensive data suggest that lifestyle aspects do not differ among the study groups, and thus do not contribute towards explaining any of the differences observed in the sperm functional parameters, leading us to conclude that these factors are not determinant for the fertility state of this particular cohort. Regarding reproductive health a similar pattern was observed, except for pre-existing urogenital infections, varicocele and urogenital anomalies, more prevalent in ID patients. Indeed, pre-existing urogenital infections and varicocele were significantly and negatively correlated with sperm viability, total motility, and chromatin integrity, in accordance with previous studies [84,85,86].

Contrarily to our observations, occupational (and environmental) exposures were previously associated with decreased sperm quality [87,88,89,90]. Yet, no studies were found using the three study groups on which the present work is focused, a fact that might justify the different results. Therefore, the available information suggests that these exposures in a professional context, although possibly impacting fertility, do not fully explain ID or UMI etiology.

Importantly the network of problems surrounding infertility are multiple, ranging from psychological to socio-economic, and these are often disregarded. In fact, while some reports suggest that infertility is a possible trigger of psychological difficulties in couples undergoing ART [91], there are others claiming that stress and anxiety can be behind the infertility status [92], making it difficult to understand the cause-consequence dynamics. The pertinence of this topic led us to include it in our analysis. Nevertheless, although patients that have/had depression have 4 times higher chance of being in the UMI group than in the CTRL (OR = 0.256), when analyzing the HADS results, the symptoms of anxiety and depression did not differ among the 3 study groups, and in principle cannot explain their different fertility status (Table S5).

As noted, to evaluate sperm functionality we did not only focus on the parameters routinely assessed but also on others that are able to provide more information on sperm function, and that have been previously suggested as good predictors of fertility capacity [11,14]. For the parameters routinely evaluated (motility, viability, and morphology), the lower values in the ID group were not surprising (Table 1, Figure S6) and are in accordance with previous reports [81,93], since these are typical features of ID patients, whose seminal quality can vary from oligo to astheno, teratozoospermic or even present the 3 abnormalities (oligoasthenoteratozoospermic), contrarily to CTRL or UMI patients, which have a normal seminal analysis [94,95]. In fact, in general, our data suggests that the UMI patient’s sperm presented a similar functional profile to that of the control group, also mirrored in statistical differences when compared to the ID group (Table 1, Figure 2 and Figure S6).

As we knew that the classification of ID and UMI patients relied essentially upon parameters evaluated in the seminal analysis, it was expected that the differences in our patients would be at more detailed levels. It is known that there are several processes crucial for spermatozoa competence [96], such as chromatin integrity [97], capacitation, acrosome reaction [98,99] or mitochondrial functionality [100,101,102,103], justifying the inclusion of the evaluation of these processes in our study. Chromatin status was only affected in ID patients (Figure 2 and Figure S6), although previous reports have shown a decrease in chromatin condensation or an increase in DNA fragmentation not only in idiopathic [104,105,106,107], but also unexplained infertile patients [108,109,110]. However, it must be considered that the methods and the studied groups were different from those used in our work, factors that might justify the different results among studies.

Additionally, in the present study, a lower percentage of capacitated cells was observed in the ID group, when compared to the CTRL group and the UMI group (Figure 2 and Figure S6). The lack of differences in this process between UMI and CTRL patients was previously reported in a study where hyperactivation, a hallmark of the capacitation process, was evaluated [111]. To our knowledge, no further studies on this topic were developed in ID or UMI patients, except for proteomic studies that have identified differentially expressed proteins in UMI patients whose functions might interfere with capacitation, acrosome reaction and gamete interaction [18], as we will further discuss. On the other hand, acrosome integrity seemed to be similar among the study groups (Figure 2 and Figure S6). Accordingly, a previous study reported no differences regarding the acrosome status when assessed in CTRL and UMI patients’ sperm [111].

Another novelty in our study was the evaluation of the mitochondrial (dys)function. Described as tightly correlated with sperm function, mitochondria establish their unique role in the sperm cell mainly through the involvement in metabolism and oxidative stress-related processes [101,103]. After evaluation of the MMP and ROS production, important indicators of mitochondrial functionality [112,113,114,115], it was observed that the MMP was affected in the ID group, when compared to CTRL group (Figure 3 and Figure S6). This, together with the observed trend to higher ROS levels in the former group, suggest that mitochondrial alterations might play a role in the (dys)function of gametes from ID patients. Our observations are in line with some studies that showed an increase in ROS levels in ID patients [116,117,118], suggesting that those patients have a compromised mitochondrial functionality. Interestingly, in the UMI group, the MMP was significantly lower than in the CTRL (Figure S6), and this the first functional aspect that seemed to distinguish these two groups. On the other hand, the pattern in terms of ROS production was very similar to that of the CTRL, in contradiction to some studies in which an increase in ROS production was also reported in UMI patients [95]. Yet, one should mention that these results were obtained using different probes to access cellular ROS (and not mitochondrial) and different cohorts.

As mentioned, several infertile couples ended up resorting to assisted reproductive techniques, in which is possible to evaluate fertilization and subsequent embryo development steps. The success or failure of those steps can foresee the fertilization capacity. Herein, after analyzing the fertilization and embryo development rate as well as biochemical pregnancy, no differences were found between the study groups, making it impossible to understand which might be the functional parameters more associated with the fertilizing potential, as was our initial intention.

Focusing on the proteomic analysis, the fact that only 14 proteins can distinguish CTRL from UMI patients shows that these patients’ proteomes have a similar profile, having fewer proteins that differ between them than with the ID proteome, confirming our observations in these patients’ sperm functional evaluation.

Focusing on the characterization of proteins associated with male infertility, *protein processing in the endoplasmic reticulum* was found to be the most significant KEGG pathway with *proteolysis* being a significant GO term (Figure 6). This suggests that a stress response, particularly related to endoplasmic reticulum (ER), might be undergoing in these patients’ sperm cells, which is in accordance with the observed trend of increased oxidative stress in the infertile patient groups. Accordingly, ROS-based ER stress was previously shown in animal models with fertility problems [119].

Additionally, we found 6 proteins differentially expressed between CTRL and both infertile groups (ID and UMI), all downregulated in the infertile groups compared to CTRL: mitochondrial citrate synthase (CISY), Dynein axonemal intermediate chain 1(DNAI1), EF-hand calcium-binding domain-containing protein 6 (EFCB6), Golgi-associated RAB2 interactor protein 3 (GAR3), Leucine-rich repeat-containing protein 37B (LR37B) and Sperm equatorial segment protein 1 (SPESP). Overall, the existing literature support a role of these proteins on processes ranging from spermatogenesis [62,120,121,122] to membrane rearrangements [54,123,124], sperm motility [52,125,126,127], acrosome reaction [128,129], gametes interaction, fertilizing capacity and pregnancy success [130,131,132] or oxidative stress [133,134,135,136], being their downregulation easily correlated with defects in those processes and having the potential to impact fertility. Moreover, as some of these 6 proteins, such as CYS and DNAI1 (when absent, altered or downregulated), were reported to be related to decreased motility, we looked deeper into our results and found that only 5 of our 29 infertile patients (ID and UMI) were asthenozoospermic, suggesting that there might be other aspects to be considered in the infertility panorama beyond motility. Worth mentioning, more than an individual effect, we suggest that the cumulative effect of the downregulation of these 6 proteins, so important for sperm vital processes, mainly energy production, motility and gametes interaction, may result in an infertile scenario.

We further analyzed the data comparing CTRL vs. ID + UMI and found that MYC (Myc proto-oncogene protein) was the transcription factor associated with more proteins differentially expressed between control and infertile individuals (Figure 6a). This factor has been described to be involved in cell proliferation, including spermatogonial stem cell proliferation [137,138] and to be associated with Sertoli cells maturity acquisition [139,140,141]. MYC was also found relevant for transcription regulation of differentially expressed seminal proteins between fertile men and bilateral varicocele patients, known to have fertility issues [20]. Importantly, MYC is present in human sperm, being involved in sperm-egg fusion processes [142], in line with our observations by GO enrichment analysis (Figure 6d). Hence, this transcription factor has a role not only in spermatogenesis and its regulation, but also in sperm-oocyte interaction and, alterations at this level might have consequences in terms of the (in)fertility state. We also found 3 relevant clusters of proteins through DAVID analysis, that are altered in ID patients, which might indicate a role of annexins [19]), serpins [19,143,144,145] and protein refolding/stress response [119,146], on the fertility state.

Focusing on the comparison among ID and UMI patients, which we consider particularly relevant, we found 8 proteins only altered between them (corresponding to the group of 8 proteins in the IDvsUMI group in Figure 5 and Figure 9), hence having a better chance to be related with the different etiology of these two types of infertility). From these only Acrosin (ACRO) and Sperm acrosome membrane-associated protein 4 (SACA4) were significantly downregulated in ID patients compared to UMI, being all the other upregulated in ID sperm (Figure 9). Globally, these proteins were associated with crucial sperm functions such as motility [49,52,59,66,75], membrane rearrangements [64,65], acrosome reaction [56], gamete interaction, fertilization process [54,55,63], and also metabolism (especially glucose metabolism) [57,58,147], mitochondrial function [50] and oxidative stress [51] and might also play a role on spermatogenesis [57,61,62,68,69] (Table 3). Yet, although for some of these proteins an association with an infertility state is easily drawn, for others the role of their altered expression on infertility is not completely clear. It is however, worth mentioning that by the random forest analysis, SACA4 and LAMB2 were the 2 factors with a higher chance of clearly distinguish ID and UMI patients, likely by acrosome integrity/reaction-related processes, being more relevant than the functional parameters considered in this study (Figure S4).

Additionally, we found that BACH2 was the most enriched transcription factor associated with most of the proteins differentially expressed between ID and UMI patients (Figure 8a). BACH2 is known to induce apoptosis in response to oxidative stress when activated by phosphorylation [148,149]. Although apoptosis was not evaluated, our results showing increased levels of chromatin damage and increased mitochondrial dysfunction in ID patients seems to be in agreement with this factor function. Furthermore, this is also in accordance with *response to hydrogen peroxide* being found as a relevant GO term (Figure 8b,c). Besides that, DAVID cluster analysis revealed a cluster of proteins related to Glycosidase activity as relevant (1.38 enrichment score). Neutral α-glucosidase AB (GANAB) present in this cluster, described to be associated with epidydimal maturation [150], was positively correlated with sperm DNA Fragmentation Index (DFI; [151], being observed to be overexpressed in ID patients (comparing to CTRL). Furthermore, as the increased activity of β-galactosidase-1 like protein (GLB1L) is a known marker of senescence already observed in testicular tissue [152], it might be suggested that its downregulation in ID patients could be a sign of sperm senescence and dysfunctional spermatogenesis.

Overall, the present study provided new information that allows distinguishing ID from UMI infertile men. Our integrated analysis indicates that aspects such as acrosome integrity, as well as metabolic elements and oxidative stress-related processes, are the ones that better differentiate the patient groups, deserving further attention in future studies. In general, it seems that UMI patients, contrarily to ID, have a better maintenance of the acrosome integrity, as seen by the proteomic data with the downregulation of LAMB2, MPI and STA10 proteins. However, these alterations were not confirmed in the functional analysis, probably because the accuracy of the relevant methods is different. It will be important to further address this aspect to confirm these observations and ascertain if they occur to avoid a premature acrosome reaction, or if they will compromise the acrosome reaction and further fertilization. On the other hand, the opposite seems to occur in ID patients as observed by the downregulation of proteins such Acrosin and SACA4. These suggest that the efficacy of the acrosome reaction, and further interaction between gametes and fertilizing ability, might be compromised in ID, also corroborated by the indication obtained from the fertilization rates, lower in ID. Similarly, the metabolic alterations identified seem to hamper ID patient sperm samples, with the more obvious consequences in terms of motility. Nonetheless, it is important to highlight that for UMI patients, an unexplained factor on the female side might also have a role in terms of couple infertility, although this investigation was out of the scope of the present research, warranting further studies.

## Figures and Tables

**Table 1:** Total motility, viability, and normal morphology in the three study groups. Data are presented by mean ± SEM and the number of samples is indicated in brackets; ***—*p* < 0.001 in comparison to CTRL; ^###^—*p* < 0.001 in comparison to ID. CTRL—healthy controls, ID—idiopathic infertile men, and UMI—unexplained infertile men).

Parameter	CTRL	ID	UMI	*p* Value
**Total motility (%)**	79.95 ± 0.73 (401)	52.13 ± 1.98 *** (194)	79.83 ± 1.41 ^###^ (103)	<0.001
**Viability (%)**	86.09 ± 0.61 (401)	66.31 ± 1.66 *** (194)	84.71 ± 1.22 ^###^ (103)	<0.001
**Normal morphology (%)**	2.30 ± 0.11 (376)	0.99 ± 0.12 *** (178)	2.47 ± 0.22 ^###^ (97)	<0.001

**Table 2:** Assisted Reproductive Techniques (ART) outcomes among the three study groups. Results are presented as mean ± SEM, and the number of experiments is indicated in brackets. Only couples in which the embryo transfer was successful were assessed for biochemical pregnancy, from which 24 (16 + 5 + 3) resulted to be positive. CTRL—healthy men, ID—idiopathic infertile men, UMI—unexplained infertile men.

ART Outcomes	CTRL	ID	UMI	*p* Value
**Fertilization rate (**123**)**	0.62 ± 0.04	0.57 ± 0.05	0.61 ± 0.07	0.786
**Embryonic development rate (**123**)**	0.71 ± 0.04	0.76 ± 0.06	0.79 ± 0.06	0.110
**Embryonic transfer rate (**123**)**	0.18 ± 0.03	0.21 ± 0.05	0.29 ± 0.09	0.917
**Biochemical pregnancy (**60**)**	16 (47.1%)	5 (35.7%)	3 (25%)	0.379

**Table 3:** Proteins differentially expressed only between ID and UMI.

Protein ID (Uniprot)	Name	ID/UMI	Biological Process Source UniProt	Function (Sperm or Male Reproductive Tract)
**P10323**	Acrosin (ACRO)	0.79	Not applicable	Important acrosomal protease released during acrosome reaction and necessary for the penetration of the zona pellucida [45,46,47]; ACRO knockout mice were fertile and capable of penetrating the zona pellucida [48]; Acrosin activity was seen decreased in infertile men whose female partners had normal gynaecological evaluation compared to fertile men (who had fathered at least one child and had normal routine semen analysis), being positively correlated with sperm motility [49]; Healthy donors with moderate or high sperm MMP have higher acrosin activity compared to men with low sperm MMP [50]; Acrosin activity decreased with oxidative stress [51]; ACRO was downregulated in asthenozoospermic patients compared to healthy donors, although it was not specified if the patients were idiopathic [52]; ACRO levels were higher in fertile men than in UMI and oligoszoospermic patients, although no significant difference was found between UMI and oligozoospermic patients [53];
**Q8TDM5**	Sperm acrosome membrane-associated protein 4 (SACA4)	0.79	Not applicable	Present on the sperm raft at the acrosome membrane, being involved in sperm-egg recognition [54]; SACA4 inhibition hampered the binding and fusion of human sperm with zona-free hamster eggs [55].
**P55268**	Laminin subunit beta-2 (LAMB2)	2.46	Mediate the attachment, migration and organization of cells into tissues during embryonic development by interacting with other extracellular matrix components.	Is present in cumulus cells and was observed to induce acrosome reaction in human sperm, in a process probably mediated by Src kinases, leading to proteasome activation and calcium influx [56]; UMI patients (categorized similar to ours) overexpressed LAMB2 compared to fertile men [19].
**P34949**	Mannose-6-phosphate isomerase (MPI)	1.45	Catalyzes the interconversion of fructose-6-phosphate and mannose-6-phosphate and is critical for the supply of D-mannose derivatives	Is expressed in mouse testis, being overexpressed in round spermatids and spermatocytes compared to spermatogonia and spermatozoa, being involved in 2-keto-3-deoxy-D-glycero-D-galacto-nononic acid (KDN) synthesis and/or the glycosylation of important factors in round spermatids [57]; MPI knockout in mice was shown embryonically lethal, due to mannose accumulation, inhibiting glucose metabolism and causing a depletion of intracellular ATP [58]; MPI was shown up-regulated in low motility compared with high motility normozoospermic samples [59]; MPI was shown downregulated in UMI patients, compared to fertile men [19].
**P17858**	ATP-dependent 6-phosphofructokinase liver type (PFKAL)	1.99	Catalyses the first committing step of glycolysis, the phosphorylation of D-fructose 6-phosphate [60]	It is highly expressed in spermatogonia [61]; PFKAL activity is decreased in spermatids compared to spermatocytes and increased in spermatozoa compared to spermatids [62]; ATP-dependent 6-phosphofructokinase muscle type was found overexpressed in sperm leading to IVF failure compared with sperm with IVF success [63].
**Q9Y365**	START domain-containing protein 10 (STA10)	1.85	Phospholipid transfer protein known to bind specifically to phosphatidylcholine and phosphatidylethmine, it can interact with the plasm membrane both in vitro and in vivo [64,65].	Not applicable
**P02787**	Serotransferrin (TRFE)	2.10	Iron binding transport proteins responsible for the transport of iron from sites of absorption and heme degradation to those of storage and utilization. Serum transferrin may also have a further role in stimulating cell proliferation. This protein has antimicrobial activity.	Was identified in boar and human seminal plasma [66,67]; Sertoli cells are known to produce TRFE, dependent on hormonal control, hence having an important role in spermatogenesis [68,69]; TRFE levels in the seminal fluid were not significantly different between the fertile men and ID patients but still associated with low sperm quality and sperm necrosis, especially in leukocytospermia and varicocele [69]; TRFE was not found in UMI patients’ sperm while being present in fertile men [19].
**P55060**	Exportin-2 (XPO2)	1.48	This protein is expressed in several types of cancers including ovarian, thyroid and testicular germ cells tumours and correlated with poor cancer outcomes [70,71,72].	XPO2 is known to induce the expression of pro-oncogenic genes [73]; XPO2 was shown downregulated in UMI patients compared to fertile males [74]; Asthenozoospermic patients shown XPO2 downregulated, compared to fertile men (not specified if the patients were idiopathic) [20,75].

## Data Availability

The data underlying this article are available in the article and in its online supplementary material. Proteomics data is available at a public repository.

## Supplementary Materials

The following supporting information can be downloaded at: https://www.mdpi.com/article/10.3390/biom13101462/s1, Figure S1: Correlation between sperm functional parameters and patients’ health aspects among the three study groups; Figure S2: Cluster analysis on proteins differentially expressed between CTRL and infertile patients using DAVID bioinformatics; Figure S3: Random forest analysis for sperm functionality parameters (concentration, viability, total motility, normal morphology, chromatin status and acrosome status) and expression of the 6 differentially expressed proteins between CTRL and infertile patients; Figure S4: Cluster analysis on proteins differentially expressed between ID and UMI patients using DAVID bioinformatics; Figure S5: Random forest analysis for sperm functionality parameters (concentration, viability, total motility, normal morphology, chromatin status and acrosome status) and expression of the 6 differentially expressed proteins between ID and UMI patients; Figure S6: Sperm functional parameters variation among the three study groups; Table S1: Study cohort characterization; Table S2: Percentage of patients presenting conditions known to affect fertility in the three study groups; Table S3: Exposure to paints, solvents, pesticides, metals, high temperatures, low temperatures, radiation and dust among the three study groups; Table S4: Consumption of alcohol and tobacco among the three study groups; Table S5: Hospital Anxiety and Depression Score (HADS) for anxiety and depression on the three study groups; Supplementary File S1: Differential proteomics analysis of sperm cells from the three study groups. Furthermore, the mass spectrometry proteomics data have been deposited to the ProteomeXchange Consortium via the PRIDE partner repository with the dataset identifier PXD0402925 and is available for reviewers (Username: reviewer_pxd040292@ebi.ac.uk; Password: bz7k6weK).

## References

[1] Salonia A., Bettocchi C., Capogrosso P., Carvalho J., Corona G., Hatzichristodoulou G., Jones T.H., Kadioglu A., Martinez-Salamanca J.I., Minhas S. EAU Guidelines. Proceedings of the EAU Annual Congress.

[2] Dyer S.J., Patel M. (2012). The economic impact of infertility on women in developing countries—A systematic review. Facts Views Vis. Obgyn..

[3] Kawwass J.F., Penzias A.S., Adashi E.Y. (2021). Fertility-a human right worthy of mandated insurance coverage: The evolution, limitations, and future of access to care. Fertil. Steril..

[4] Dooley M., Dineen T., Sarma K., Nolan A. (2014). The psychological impact of infertility and fertility treatment on the male partner. Hum. Fertil..

[5] Agarwal A., Mulgund A., Hamada A., Chyatte M.R. (2015). A unique view on male infertility around the globe. Reprod. Biol. Endocrinol..

[6] Rusz A., Pilatz A., Wagenlehner F., Linn T., Diemer T., Schuppe H., Lohmeyer J., Hossain H., Weidner W. (2012). Influence of urogenital infections and inflammation on semen quality and male fertility. World J. Urol..

[7] Diamanti-Kandarakis E., Bourguignon J.-P., Giudice L.C., Hauser R., Prins G.S., Soto A.M., Zoeller R.T., Gore A.C. (2009). Endocrine-disrupting chemicals: An Endocrine Society scientific statement. Endocr. Rev..

[8] Minhas S., Bettocchi C., Boeri L., Capogrosso P., Carvalho J., Cilesiz N.C., Cocci A., Corona G., Dimitropoulos K., Gül M. (2021). European Association of Urology Guidelines on Male Sexual and Reproductive Health: 2021 Update on Male Infertility. Eur. Urol..

[9] Pierik F.H., Van Ginneken A.M., Dohle G.R., Vreeburg J.T., Weber R.F. (2000). The advantages of standardized evaluation of male infertility. Int. J. Androl..

[10] Hamada A., Esteves S.C., Agarwal A. (2011). Unexplained male infertility: Potential causes and management. Hum. Androl..

[11] Arafa M., Agarwal A., Majzoub A., Panner Selvam M.K., Baskaran S., Henkel R., Elbardisi H. (2020). Efficacy of antioxidant supplementation on conventional and advanced sperm function tests in patients with idiopathic male infertility. Antioxidants.

[12] Hamada A., Esteves S.C., Nizza M., Agarwal A. (2012). Unexplained male infertility: Diagnosis and management. Int. Braz. J. Urol..

[13] Collins J.A., Crosignani P.G. (1992). Unexplained infertility: A review of diagnosis, prognosis, treatment efficacy and management. Int. J. Gynaecol. Obs..

[14] Hamada A., Esteves S.C., Agarwal A. (2011). The role of contemporary andrology in unraveling the mystery of unexplained male infertility. Open Reprod. Sci. J..

[15] Amaral A., Castillo J., Ramalho-Santos J., Oliva R. (2014). The combined human sperm proteome: Cellular pathways and implications for basic and clinical science. Hum. Reprod. Update.

[16] Moscatelli N., Lunetti P., Braccia C., Armirotti A., Pisanello F., De Vittorio M., Zara V., Ferramosca A. (2019). Comparative proteomic analysis of proteins involved in bioenergetics pathways associated with human sperm motility. Int. J. Mol. Sci..

[17] Shen S., Wang J., Liang J., He D. (2013). Comparative proteomic study between human normal motility sperm and idiopathic asthenozoospermia. World J. Urol..

[18] Xu W., Hu H., Wang Z., Chen X., Yang F., Zhu Z., Fang P., Dai J., Wang L., Shi H. (2012). Proteomic characteristics of spermatozoa in normozoospermic patients with infertility. J. Proteom..

[19] Panner Selvam M.K., Agarwal A., Pushparaj P.N., Baskaran S., Bendou H. (2019). Sperm proteome analysis and identification of fertility-associated biomarkers in unexplained male infertility. Genes.

[20] Panner Selvam M.K., Agarwal A., Baskaran S. (2019). Proteomic analysis of seminal plasma from bilateral varicocele patients indicates an oxidative state and increased inflammatory response. Asian J. Androl..

[21] Kliesch S. (2014). Diagnosis of male infertility: Diagnostic work-up of the infertile man. Eur. Urol. Suppl..

[22] WHO Organization (2010). WHO Laboratory Manual for the Examination and Processing of Human Semen.

[23] Portela J., Tavares R., Mota P., Ramalho-Santos J., Amaral S. (2015). High glucose concentrations per se do not adversely affect human sperm function in vitro. Reproduction.

[24] Zigmond A.S., Snaith R.P. (1983). The hospital anxiety and depression scale. Acta Psychiatr Scand..

[25] Pais-Ribeiro J., Silva I., Ferreira T., Martins A., Meneses R., Baltar M. (2007). Validation study of a Portuguese version of the Hospital Anxiety and Depression Scale. Psychol. Health Med..

[26] Chaves C., Canavarro M.C., Moura-Ramos M. (2019). The Role of Dyadic Coping on the Marital and Emotional Adjustment of Couples With Infertility. Fam. Process.

[27] Djukanovic I., Carlsson J., Årestedt K. (2017). Is the Hospital Anxiety and Depression Scale (HADS) a valid measure in a general population 65–80 years old? A psychometric evaluation study. Health Qual. Life Outcomes.

[28] Sousa A.P.M., Tavares R.S., Velez de la Calle J.F., Figueiredo H., Almeida V., Almeida-Santos T., Ramalho-Santos J. (2009). Dual use of Diff-Quik-like stains for the simultaneous evaluation of human sperm morphology and chromatin status. Hum. Reprod..

[29] Ramalho-Santos J., Amaral A., Sousa A.P., Rodrigues A.S., Martins L., Baptista M., Mota P.C., Tavares R., Amaral S., Gamboa S. (2007). Probing the structure and function of mammalian sperm using optical and fluorescence microscopy. Mod. Res. Educ. Top. Microsc..

[30] Amaral S., Redmann K., Sanchez V., Mallidis C., Ramalho-Santos J., Schlatt S. (2013). UVB irradiation as a tool to assess ROS-induced damage in human spermatozoa. Andrology.

[31] Sousa M.I., Amaral S., Tavares R.S., Paiva C., Ramalho-Santos J. (2014). Concentration-dependent Sildenafil citrate (Viagra) effects on ROS production, energy status, and human sperm function. Syst. Biol. Reprod. Med..

[32] Tavares R., Silva A., Lourenço B., Almeida-Santos T., Sousa A., Ramalho-Santos J. (2013). Evaluation of human sperm chromatin status after selection using a modified Diff-Quik stain indicates embryo quality and pregnancy outcomes following in vitro fertilization. Andrology.

[33] Anjo S.I., Santa C., Manadas B. (2015). Short GeLC-SWATH: A fast and reliable quantitative approach for proteomic screenings. Proteomics.

[34] Collins B.C., Gillet L.C., Rosenberger G., Röst H.L., Vichalkovski A., Gstaiger M., Aebersold R. (2013). Quantifying protein interaction dynamics by SWATH mass spectrometry: Application to the 14-3-3 system. Nat. Methods.

[35] Gillet L.C., Navarro P., Tate S., Röst H., Selevsek N., Reiter L., Bonner R., Aebersold R. (2012). Targeted data extraction of the MS/MS spectra generated by data-independent acquisition: A new concept for consistent and accurate proteome analysis. Mol. Cell. Proteom..

[36] Sennels L., Bukowski-Wills J.-C., Rappsilber J. (2009). Improved results in proteomics by use of local and peptide-class specific false discovery rates. BMC Bioinform..

[37] Tang W.H., Shilov I.V., Seymour S.L. (2008). Nonlinear fitting method for determining local false discovery rates from decoy database searches. J. Proteome Res..

[38] Lambert J.-P., Ivosev G., Couzens A.L., Larsen B., Taipale M., Lin Z.-Y., Zhong Q., Lindquist S., Vidal M., Aebersold R. (2013). Mapping differential interactomes by affinity purification coupled with data-independent mass spectrometry acquisition. Nat. Methods.

[39] Anjo S.I., Simões I., Castanheira P., Grãos M., Manadas B. (2019). Use of recombinant proteins as a simple and robust normalization method for untargeted proteomics screening: Exhaustive performance assessment. Talanta.

[40] Perez-Riverol Y., Bai J., Bandla C., García-Seisdedos D., Hewapathirana S., Kamatchinathan S., Kundu D.J., Prakash A., Frericks-Zipper A., Eisenacher M. (2022). The PRIDE database resources in 2022: A hub for mass spectrometry-based proteomics evidences. Nucleic Acids Res.

[41] Mi H., Muruganujan A., Huang X., Ebert D., Mills C., Guo X., Thomas P.D. (2019). Protocol Update for large-scale genome and gene function analysis with the PANTHER classification system (v.14.0). Nat. Protoc..

[42] Huang d.W., Sherman B.T., Lempicki R.A. (2009). Systematic and integrative analysis of large gene lists using DAVID bioinformatics resources. Nat. Protoc..

[43] Pathan M., Keerthikumar S., Ang C.S., Gangoda L., Quek C.Y., Williamson N.A., Mouradov D., Sieber O.M., Simpson R.J., Salim A. (2015). FunRich: An open access standalone functional enrichment and interaction network analysis tool. Proteomics.

[44] Chong J., Wishart D.S., Xia J. (2019). Using MetaboAnalyst 4.0 for Comprehensive and Integrative Metabolomics Data Analysis. Curr. Protoc. Bioinform..

[45] Meizel S., Mukerji S.K. (1975). Proacrosin from rabbit epididymal spermatozoa: Partial purification and initial biochemical characterization. Biol. Reprod..

[46] Hirose M., Honda A., Fulka H., Tamura-Nakano M., Matoba S., Tomishima T., Mochida K., Hasegawa A., Nagashima K., Inoue K. (2020). Acrosin is essential for sperm penetration through the zona pellucida in hamsters. Proc. Natl. Acad. Sci. USA.

[47] Urch U.A., Wardrip N.J., Hedrick J.L. (1985). Proteolysis of the zona pellucida by acrosin: The nature of the hydrolysis products. J. Exp. Zool..

[48] Baba T., Azuma S., Kashiwabara S., Toyoda Y. (1994). Sperm from mice carrying a targeted mutation of the acrosin gene can penetrate the oocyte zona pellucida and effect fertilization. J. Biol. Chem..

[49] Cui Y.H., Zhao R.L., Wang Q., Zhang Z.Y. (2000). Determination of sperm acrosin activity for evaluation of male fertility. Asian J. Androl..

[50] Zhang G., Yang W., Zou P., Jiang F., Zeng Y., Chen Q., Sun L., Yang H., Zhou N., Wang X. (2019). Mitochondrial functionality modifies human sperm acrosin activity, acrosome reaction capability and chromatin integrity. Hum. Reprod..

[51] Zalata A., El-Samanoudy A.Z., Shaalan D., El-Baiomy Y., Mostafa T. (2015). In vitro effect of cell phone radiation on motility, DNA fragmentation and clusterin gene expression in human sperm. Int. J. Fertil. Steril..

[52] Guo Y., Jiang W., Yu W., Niu X., Liu F., Zhou T., Zhang H., Li Y., Zhu H., Zhou Z. (2019). Proteomics analysis of asthenozoospermia and identification of glucose-6-phosphate isomerase as an important enzyme for sperm motility. J. Proteom..

[53] Mohsenian M., Syner F.N., Moghissi K.S. (1982). A study of sperm acrosin in patients with unexplained infertility. Fertil. Steril..

[54] Nixon B., Mitchell L.A., Anderson A.L., McLaughlin E.A., O’bryan M.K., Aitken R.J. (2011). Proteomic and functional analysis of human sperm detergent resistant membranes. J. Cell Physiol..

[55] Shetty J., Wolkowicz M.J., Digilio L.C., Klotz K.L., Jayes F.L., Diekman A.B., Westbrook V.A., Farris E.M., Hao Z., Coonrod S.A. (2003). SAMP14, a novel, acrosomal membrane-associated, glycosylphosphatidylinositol-anchored member of the Ly-6/urokinase-type plasminogen activator receptor superfamily with a role in sperm-egg interaction. J. Biol. Chem..

[56] Tapia S., Rojas M., Morales P., Ramirez M.A., Diaz E.S. (2011). The laminin-induced acrosome reaction in human sperm is mediated by Src kinases and the proteasome. Biol. Reprod..

[57] Davis J.A., Wu X.H., Wang L., DeRossi C., Westphal V., Wu R., Alton G., Srikrishna G., Freeze H.H. (2002). Molecular cloning, gene organization, and expression of mouse Mpi encoding phosphomannose isomerase. Glycobiology.

[58] DeRossi C., Bode L., Eklund E.A., Zhang F., Davis J.A., Westphal V., Wang L., Borowsky A.D., Freeze H.H. (2006). Ablation of mouse phosphomannose isomerase (Mpi) causes mannose 6-phosphate accumulation, toxicity, and embryonic lethality. J. Biol. Chem..

[59] Martin-Hidalgo D., Serrano R., Zaragoza C., Garcia-Marin L.J., Bragado M.J. (2020). Human sperm phosphoproteome reveals differential phosphoprotein signatures that regulate human sperm motility. J. Proteom..

[60] Yi W., Clark P.M., Mason D.E., Keenan M.C., Hill C., Goddard III W.A., Peters E.C., Driggers E.M., Hsieh-Wilson L.C. (2012). PFK1 glycosylation is a key regulator of cancer cell growth and central metabolic pathways. Science.

[61] Park Y.-J., Pang M.-G. (2021). Mitochondrial functionality in male fertility: From spermatogenesis to fertilization. Antioxidants.

[62] Bajpai M., Gupta G., Setty B.S. (1998). Changes in carbohydrate metabolism of testicular germ cells during meiosis in the rat. Eur. J. Endocrinol..

[63] Zhu Y., Wu Y., Jin K., Lu H., Liu F., Guo Y., Yan F., Shi W., Liu Y., Cao X. (2013). Differential proteomic profiling in human spermatozoa that did or did not result in pregnancy via IVF and AID. Proteom.–Clin. Appl..

[64] Olayioye M.A., Hoffmann P., Pomorski T., Armes J., Simpson R.J., Kemp B.E., Lindeman G.J., Visvader J.E. (2004). The phosphoprotein StarD10 is overexpressed in breast cancer and cooperates with ErbB receptors in cellular transformation. Cancer Res..

[65] Olayioye M.A., Vehring S., Müller P., Herrmann A., Schiller J., Thiele C., Lindeman G.J., Visvader J.E., Pomorski T. (2005). StarD10, a START domain protein overexpressed in breast cancer, functions as a phospholipid transfer protein. J. Biol. Chem..

[66] Collodel G., Signorini C., Nerucci F., Gambera L., Iacoponi F., Moretti E. (2021). Semen Biochemical Components in Varicocele, Leukocytospermia, and Idiopathic Infertility. Reprod. Sci..

[67] González-Cadavid V., Martins J.A., Moreno F.B., Andrade T.S., Santos A.C., Monteiro-Moreira A.C., Moreira R.A., Moura A.A. (2014). Seminal plasma proteins of adult boars and correlations with sperm parameters. Theriogenology.

[68] Skinner M.K., Griswold M.D. (1980). Sertoli cells synthesize and secrete transferrin-like protein. J. Biol. Chem..

[69] Skinner M.K., Griswold M.D. (1982). Secretion of testicular transferrin by cultured Sertoli cells is regulated by hormones and retinoids. Biol. Reprod..

[70] Holzer K., Drucker E., Oliver S., Winkler J., Eiteneuer E., Herpel E., Breuhahn K., Singer S. (2016). Cellular apoptosis susceptibility (CAS) is overexpressed in thyroid carcinoma and maintains tumor cell growth: A potential link to the BRAFV600E mutation. Int. J. Oncol..

[71] Jiang M.C. (2016). CAS (CSE1L) signaling pathway in tumor progression and its potential as a biomarker and target for targeted therapy. Tumour Biol..

[72] Liu C., Wei J., Xu K., Sun X., Zhang H., Xiong C. (2019). CSE1L participates in regulating cell mitosis in human seminoma. Cell Prolif..

[73] Lorenzato A., Biolatti M., Delogu G., Capobianco G., Farace C., Dessole S., Cossu A., Tanda F., Madeddu R., Olivero M. (2013). AKT activation drives the nuclear localization of CSE1L and a pro-oncogenic transcriptional activation in ovarian cancer cells. Exp. Cell Res..

[74] Lorenzato A., Martino C., Dani N., Oligschläger Y., Ferrero A.M., Biglia N., Calogero R., Olivero M., Di Renzo M.F. (2012). The cellular apoptosis susceptibility CAS/CSE1L gene protects ovarian cancer cells from death by suppressing RASSF1C. FASEB J..

[75] Amaral A., Paiva C., Attardo Parrinello C., Estanyol J.M., Ballescà J.L., Ramalho-Santos J., Oliva R. (2014). Identification of proteins involved in human sperm motility using high-throughput differential proteomics. J. Proteome Res..

[76] Hamada A., Esteves S., Agarwal A. (2012). Unexplained male infertility—Looking beyond routine semen analysis. Eur. Urol. Rev..

[77] Wang C., Swerdloff R.S. (2014). Limitations of semen analysis as a test of male fertility and anticipated needs from newer tests. Fertil. Steril..

[78] Barazani Y., Agarwal A., Sabanegh Jr E.S. (2014). Functional sperm testing and the role of proteomics in the evaluation of male infertility. Urology.

[79] Schattman G.L., Esteves S.C., Agarwal A. (2015). Unexplained Infertility: Pathophysiology, Evaluation and Treatment.

[80] Esteves S.C., Miyaoka R., Agarwal A. (2011). An update on the clinical assessment of the infertile male. Clinics.

[81] Arcaniolo D., Favilla V., Tiscione D., Pisano F., Bozzini G., Creta M., Gentile G., Fabris F.M., Pavan N., Veneziano I.A. (2014). Is there a place for nutritional supplements in the treatment of idiopathic male infertility?. Arch. Ital. Urol. Androl..

[82] Kanannejad Z., Gharesi-Fard B. (2019). Difference in the seminal plasma protein expression in unexplained infertile men with successful and unsuccessful in vitro fertilisation outcome. Andrologia.

[83] Corsini C., Boeri L., Candela L., Pozzi E., Belladelli F., Capogrosso P., Fallara G., Schifano N., Cignoli D., Ventimiglia E. (2021). Is There a Relevant Clinical Impact in Differentiating Idiopathic versus Unexplained Male Infertility?. World J. Men’s Health.

[84] Damsgaard J., Joensen U.N., Carlsen E., Erenpreiss J., Jensen M.B., Matulevicius V., Zilaitiene B., Olesen I.A., Perheentupa A., Punab M. (2016). Varicocele is associated with impaired semen quality and reproductive hormone levels: A study of 7035 healthy young men from six European countries. Eur. Urol..

[85] Santana V.P., James E.R., Miranda-Furtado C.L., de Souza M.F., Pompeu C.P., Esteves S.C., Carrell D.T., Aston K.I., Jenkins T.G., Dos Reis R.M. (2020). Differential DNA methylation pattern and sperm quality in men with varicocele. Fertil. Steril..

[86] Tahamtan S., Tavalaee M., Izadi T., Barikrow N., Zakeri Z., Lockshin R.A., Abbasi H., Nasr-Esfahani M.H. (2019). Reduced sperm telomere length in individuals with varicocele is associated with reduced genomic integrity. Sci. Rep..

[87] Wdowiak A., Skrzypek M., Stec M., Panasiuk L. (2019). Effect of ionizing radiation on the male reproductive system. Ann. Agric. Environ. Med..

[88] Ashiru O.A., Odusanya O.O. (2009). Fertility and occupational hazards: Review of the literature. Afr. J. Reprod. Health.

[89] Yucra S., Rubio J., Gasco M., Gonzales C., Steenland K., Gonzales G.F. (2006). Semen quality and reproductive sex hormone levels in Peruvian pesticide sprayers. Int. J. Occup. Environ. Health.

[90] Bonde J.P. (1992). Semen quality in welders exposed to radiant heat. Occup. Environ. Med..

[91] Pozza A., Dèttore D., Coccia M.E. (2019). Depression and anxiety in pathways of medically assisted reproduction: The role of infertility stress dimensions. Clin. Pract. Epidemiol. Ment. Health CP EMH.

[92] De Gennaro L., Balistreri S., Lenzi A., Lombardo F., Ferrara M., Gandini L. (2003). Psychosocial factors discriminate oligozoospermic from normozoospermic men. Fertil. Steril..

[93] Kumar R., Venkatesh S., Kumar M., Tanwar M., Shasmsi M., Gupta N., Sharma R., Talwar P., Dada R. (2009). Oxidative Stress and Sperm Mitochondrial Dna Mutation in Idiopathic Oligoasthenozoospermic Men. Indian J. Biochem. Biophys..

[94] Mayorga-Torres B.J., Cardona-Maya W., Cadavid Á., Camargo M. (2013). Evaluation of sperm functional parameters in normozoospermic infertile individuals. Actas Urol. Esp..

[95] Mayorga-Torres B.J.M., Camargo M., Cadavid Á., du Plessis S.S., Cardona Maya W.D. (2017). Are oxidative stress markers associated with unexplained male infertility?. Andrologia.

[96] Lewis S.E. (2007). Is sperm evaluation useful in predicting human fertility?. Reproduction.

[97] Agarwal A., Said T.M. (2003). Role of sperm chromatin abnormalities and DNA damage in male infertility. Hum. Reprod. Update.

[98] Abou-haila A., Tulsiani D.R. (2009). Signal transduction pathways that regulate sperm capacitation and the acrosome reaction. Arch. Biochem. Biophys..

[99] De Jonge C. (2017). Biological basis for human capacitation—Revisited. Hum. Reprod. Update.

[100] Amaral S., Ramalho-Santos J. (2009). Aging, mitochondria and male reproductive function. Curr. Aging Sci..

[101] Sousa A.P., Amaral A., Baptista M., Tavares R., Caballero Campo P., Caballero Peregrín P., Freitas A., Paiva A., Almeida-Santos T., Ramalho-Santos J. (2011). Not all sperm are equal: Functional mitochondria characterize a subpopulation of human sperm with better fertilization potential. PLoS ONE.

[102] Marchetti P., Ballot C., Jouy N., Thomas P., Marchetti C. (2012). Influence of mitochondrial membrane potential of spermatozoa on in vitro fertilisation outcome. Andrologia.

[103] Amaral A., Lourenço B., Marques M., Ramalho-Santos J. (2013). Mitochondria functionality and sperm quality. Reproduction.

[104] Salsabili N., Mehrsai A., Jalalizadeh B., Pourmand G., Jalaie S. (2006). Correlation of sperm nuclear chromatin condensation staining method with semen parameters and sperm functional tests in patients with spinal cord injury, varicocele, and idiopathic infertility. Urol. J..

[105] Wu W., Shen O., Qin Y., Niu X., Lu C., Xia Y., Song L., Wang S., Wang X. (2010). Idiopathic male infertility is strongly associated with aberrant promoter methylation of methylenetetrahydrofolate reductase (MTHFR). PLoS ONE.

[106] Palomba S., Falbo A., Espinola S., Rocca M., Capasso S., Cappiello F., Zullo F. (2011). Effects of highly purified follicle-stimulating hormone on sperm DNA damage in men with male idiopathic subfertility: A pilot study. J. Endocrinol. Investig..

[107] Pelliccione F., d’Angeli A., Cinque B., Falone S., Micillo A., Francavilla F., Amicarelli F., Gandini L., Francavilla S. (2011). Activation of the immune system and sperm DNA fragmentation are associated with idiopathic oligoasthenoteratospermia in men with couple subfertility. Fertil. Steril..

[108] Saleh R.A., Agarwal A., Nelson D.R., Nada E.A., El-Tonsy M.H., Alvarez J.G., Thomas A.J., Sharma R.K. (2002). Increased sperm nuclear DNA damage in normozoospermic infertile men: A prospective study. Fertil. Steril..

[109] Urdinguio R.G., Bayón G.F., Dmitrijeva M., Toraño E.G., Bravo C., Fraga M.F., Bassas L., Larriba S., Fernández A.F. (2015). Aberrant DNA methylation patterns of spermatozoa in men with unexplained infertility. Hum. Reprod..

[110] Faduola P., Kolade C.O. (2015). Sperm chromatin structure assay results in Nigerian men with unexplained infertility. Clin. Exp. Reprod. Med..

[111] Peedicayil J., Deendayal M., Sadasivan G., Shivaji S. (1997). Assessment of hyperactivation, acrosome reaction and motility characteristics of spermatozoa from semen of men of proven fertility and unexplained infertility. Andrologia.

[112] Cocuzza M., Sikka S.C., Athayde K.S., Agarwal A. (2007). Clinical relevance of oxidative stress and sperm chromatin damage in male infertility: An evidence based analysis. Int. Braz. J. Urol..

[113] Tremellen K. (2008). Oxidative stress and male infertility--a clinical perspective. Hum. Reprod. Update.

[114] Agarwal A., Allamaneni S.S. (2011). Free radicals and male reproduction. J. Indian Med. Assoc..

[115] Zorova L.D., Popkov V.A., Plotnikov E.Y., Silachev D.N., Pevzner I.B., Jankauskas S.S., Babenko V.A., Zorov S.D., Balakireva A.V., Juhaszova M. (2018). Mitochondrial membrane potential. Anal. Biochem..

[116] Pasqualotto F.F., Sharma R.K., Kobayashi H., Nelson D.R., Thomas A.J., Agarwal A. (2001). Oxidative stress in normospermic men undergoing infertility evaluation. J. Androl..

[117] Aydemir B., Onaran I., Kiziler A.R., Alici B., Akyolcu M.C. (2007). Increased oxidative damage of sperm and seminal plasma in men with idiopathic infertility is higher in patients with glutathione S-transferase Mu-1 null genotype. Asian J. Androl..

[118] Aktan G., Doğru-Abbasoğlu S., Küçükgergin C., Kadıoğlu A., Ozdemirler-Erata G., Koçak-Toker N. (2013). Mystery of idiopathic male infertility: Is oxidative stress an actual risk?. Fertil. Steril..

[119] Soni K.K., Zhang L.T., Choi B.R., Karna K.K., You J.H., Shin Y.S., Lee S.W., Kim C.Y., Zhao C., Chae H.J. (2018). Protective effect of MOTILIPERM in varicocele-induced oxidative injury in rat testis by activating phosphorylated inositol requiring kinase 1α (p-IRE1α) and phosphorylated c-Jun N-terminal kinase (p-JNK) pathways. Pharm. Biol..

[120] Amaral A., Castillo J., Estanyol J.M., Ballescà J.L., Ramalho-Santos J., Oliva R. (2013). Human sperm tail proteome suggests new endogenous metabolic pathways. Mol. Cell. Proteom..

[121] Petit F.G., Kervarrec C., Jamin S.P., Smagulova F., Hao C., Becker E., Jégou B., Chalmel F., Primig M. (2015). Combining RNA and protein profiling data with network interactions identifies genes associated with spermatogenesis in mouse and human. Biol. Reprod..

[122] de Mateo S., Castillo J., Estanyol J.M., Ballescà J.L., Oliva R. (2011). Proteomic characterization of the human sperm nucleus. Proteomics.

[123] Asano A., Selvaraj V., Buttke D.E., Nelson J.L., Green K.M., Evans J.E., Travis A.J. (2009). Biochemical characterization of membrane fractions in murine sperm: Identification of three distinct sub-types of membrane rafts. J. Cell Physiol..

[124] López-Salguero J.B., Fierro R., Michalski J.C., Jiménez-Morales I., Lefebvre T., Mondragón-Payne O., Baldini S.F., Vercoutter-Edouart A.S., González-Márquez H. (2020). Identification of lipid raft glycoproteins obtained from boar spermatozoa. Glycoconj. J..

[125] Chen L., Wen C.W., Deng M.J., Li P., Zhang Z.D., Zhou Z.H., Wang X. (2020). Metabolic and transcriptional changes in seminal plasma of asthenozoospermia patients. Biomed. Chromatogr..

[126] Pereira R., Oliveira J., Ferraz L., Barros A., Santos R., Sousa M. (2015). Mutation analysis in patients with total sperm immotility. J. Assist. Reprod. Genet..

[127] Hwang J.Y., Mannowetz N., Zhang Y., Everley R.A., Gygi S.P., Bewersdorf J., Lishko P.V., Chung J.J. (2019). Dual Sensing of Physiologic pH and Calcium by EFCAB9 Regulates Sperm Motility. Cell.

[128] Wolkowicz M.J., Shetty J., Westbrook A., Klotz K., Jayes F., Mandal A., Flickinger C.J., Herr J.C. (2003). Equatorial segment protein defines a discrete acrosomal subcompartment persisting throughout acrosomal biogenesis. Biol. Reprod..

[129] Wolkowicz M.J., Digilio L., Klotz K., Shetty J., Flickinger C.J., Herr J.C. (2008). Equatorial segment protein (ESP) is a human alloantigen involved in sperm-egg binding and fusion. J. Androl..

[130] Novak S., Smith T.A., Paradis F., Burwash L., Dyck M.K., Foxcroft G.R., Dixon W.T. (2010). Biomarkers of in vivo fertility in sperm and seminal plasma of fertile stallions. Theriogenology.

[131] Xue D., Zhang Y., Wang Y., Wang J., An F., Sun X., Yu Z. (2019). Quantitative proteomic analysis of sperm in unexplained recurrent pregnancy loss. Reprod. Biol. Endocrinol..

[132] Enoiu S.I., Nygaard M.B., Bungum M., Ziebe S., Petersen M.R., Almstrup K. (2022). Expression of membrane fusion proteins in spermatozoa and total fertilisation failure during in vitro fertilisation. Andrology.

[133] Ariga H., Takahashi-Niki K., Kato I., Maita H., Niki T., Iguchi-Ariga S.M. (2013). Neuroprotective function of DJ-1 in Parkinson’s disease. Oxid. Med. Cell Longev..

[134] Strobbe D., Robinson A.A., Harvey K., Rossi L., Ferraina C., de Biase V., Rodolfo C., Harvey R.J., Campanella M. (2018). Distinct Mechanisms of Pathogenic DJ-1 Mutations in Mitochondrial Quality Control. Front. Mol. Neurosci..

[135] Wu Y.Q., Rao M., Hu S.F., Ke D.D., Zhu C.H., Xia W. (2020). Effect of transient scrotal hyperthermia on human sperm: An iTRAQ-based proteomic analysis. Reprod. Biol. Endocrinol..

[136] Agarwal A., Panner Selvam M.K., Samanta L., Vij S.C., Parekh N., Sabanegh E., Tadros N.N., Arafa M., Sharma R. (2019). Effect of Antioxidant Supplementation on the Sperm Proteome of Idiopathic Infertile Men. Antioxidants.

[137] Kanatsu-Shinohara M., Onoyama I., Nakayama K.I., Shinohara T. (2014). Skp1-Cullin-F-box (SCF)-type ubiquitin ligase FBXW7 negatively regulates spermatogonial stem cell self-renewal. Proc. Natl. Acad. Sci. USA.

[138] Kanatsu-Shinohara M., Tanaka T., Ogonuki N., Ogura A., Morimoto H., Cheng P.F., Eisenman R.N., Trumpp A., Shinohara T. (2016). Myc/Mycn-mediated glycolysis enhances mouse spermatogonial stem cell self-renewal. Genes. Dev..

[139] Meroni S.B., Galardo M.N., Rindone G., Gorga A., Riera M.F., Cigorraga S.B. (2019). Molecular Mechanisms and Signaling Pathways Involved in Sertoli Cell Proliferation. Front. Endocrinol..

[140] Lim K., Hwang B.D. (1995). Follicle-stimulating hormone transiently induces expression of protooncogene c-myc in primary Sertoli cell cultures of early pubertal and prepubertal rat. Mol. Cell Endocrinol..

[141] Riera M.F., Regueira M., Galardo M.N., Pellizzari E.H., Meroni S.B., Cigorraga S.B. (2012). Signal transduction pathways in FSH regulation of rat Sertoli cell proliferation. Am. J. Physiol. Endocrinol. Metab..

[142] Naz R.K., Ahmad K., Kumar G. (1991). Presence and role of c-myc proto-oncogene product in mammalian sperm cell function. Biol. Reprod..

[143] Afsari M., Fesahat F., Talebi A.R., Agarwal A., Henkel R., Zare F., Gül M., Iraci N., Cannarella R., Makki M. (2022). ANXA2, SP17, SERPINA5, PRDX2 genes, and sperm DNA fragmentation differentially represented in male partners of infertile couples with normal and abnormal sperm parameters. Andrologia.

[144] Odet F., Verot A., Le Magueresse-Battistoni B. (2006). The mouse testis is the source of various serine proteases and serine proteinase inhibitors (SERPINs): Serine proteases and SERPINs identified in Leydig cells are under gonadotropin regulation. Endocrinology.

[145] Odet F., Guyot R., Leduque P., Le Magueresse-Battistoni B. (2004). Evidence for similar expression of protein C inhibitor and the urokinase-type plasminogen activator system during mouse testis development. Endocrinology.

[146] Karna K.K., Shin Y.S., Choi B.R., Kim H.K., Park J.K. (2020). The Role of Endoplasmic Reticulum Stress Response in Male Reproductive Physiology and Pathology: A Review. World J. Mens. Health.

[147] Niehues R., Hasilik M., Alton G., Körner C., Schiebe-Sukumar M., Koch H.G., Zimmer K.P., Wu R., Harms E., Reiter K. (1998). Carbohydrate-deficient glycoprotein syndrome type Ib. Phosphomannose isomerase deficiency and mannose therapy. J. Clin. Investig..

[148] Kamio T., Toki T., Kanezaki R., Sasaki S., Tandai S., Terui K., Ikebe D., Igarashi K., Ito E. (2003). B-cell-specific transcription factor BACH2 modifies the cytotoxic effects of anticancer drugs. Blood.

[149] Yoshida C., Yoshida F., Sears D.E., Hart S.M., Ikebe D., Muto A., Basu S., Igarashi K., Melo J.V. (2007). Bcr-Abl signaling through the PI-3/S6 kinase pathway inhibits nuclear translocation of the transcription factor Bach2, which represses the antiapoptotic factor heme oxygenase-1. Blood.

[150] Dacheux J.L., Gatti J.L., Dacheux F. (2003). Contribution of epididymal secretory proteins for spermatozoa maturation. Microsc. Res. Tech..

[151] Watanabe M., Roussev R., Ahlering P., Sauer R., Coulam C., Jeyendran R.S. (2009). Correlation between neutral alpha-glucosidase activity and sperm DNA fragmentation. Andrologia.

[152] Schmid N., Flenkenthaler F., Stöckl J.B., Dietrich K.G., Köhn F.M., Schwarzer J.U., Kunz L., Luckner M., Wanner G., Arnold G.J. (2019). Insights into replicative senescence of human testicular peritubular cells. Sci. Rep..

