# Supplementary material for: New Insights on Sperm Function in Male Infertility of Unknown Origin: A Multimodal Approach
Source: Biomolecules. 2023 Sep 27;13(10):1462. doi: 10.3390/biom13101462 (PMC10605211; doi:10.3390/biom13101462)

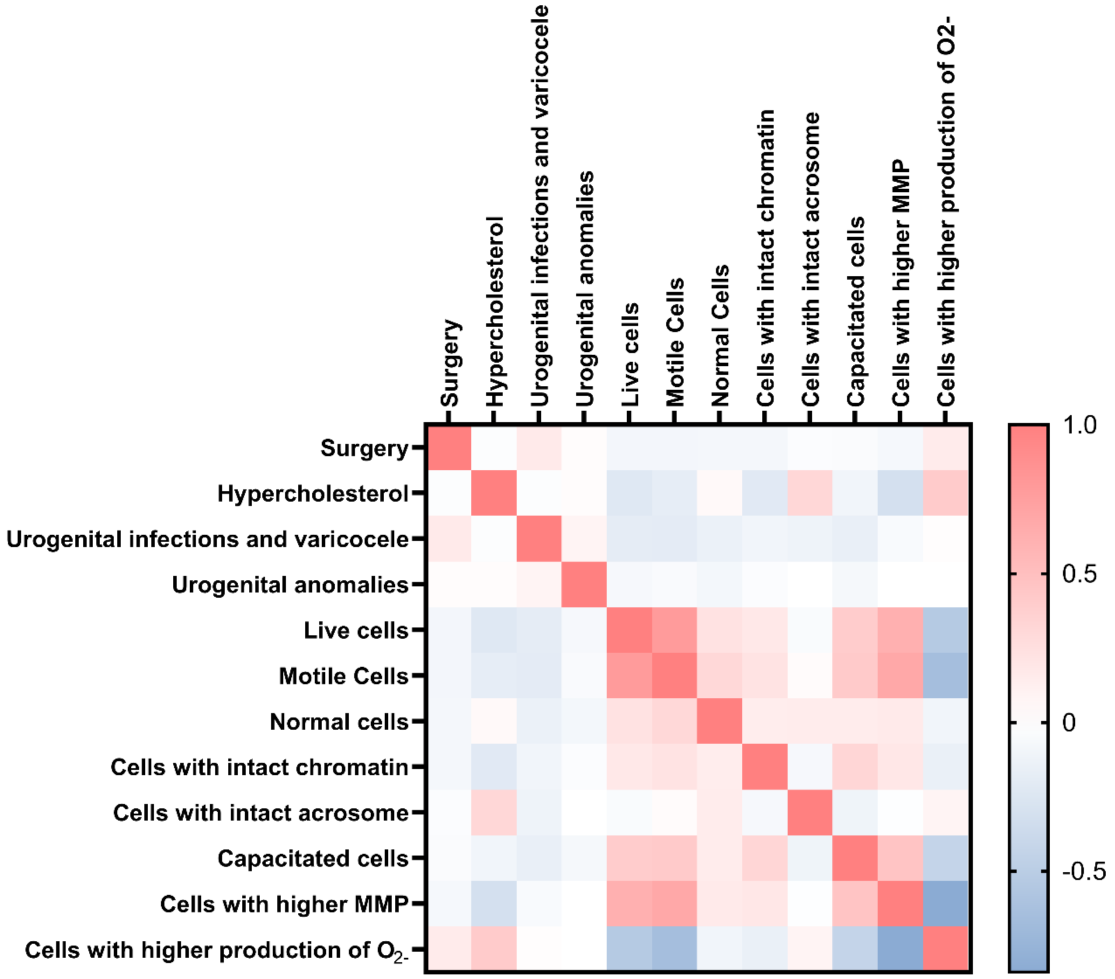

Figure S1 – Correlation between sperm functional parameters and patients' health among the three study groups (CTRL – healthy controls, ID- idiopathic male infertility, UMI- unexplained male infertility). The colour scale represents the "r" value obtained by the Spearman correlations. The red tones stand for positive correlations while the blue tones stand for negative ones

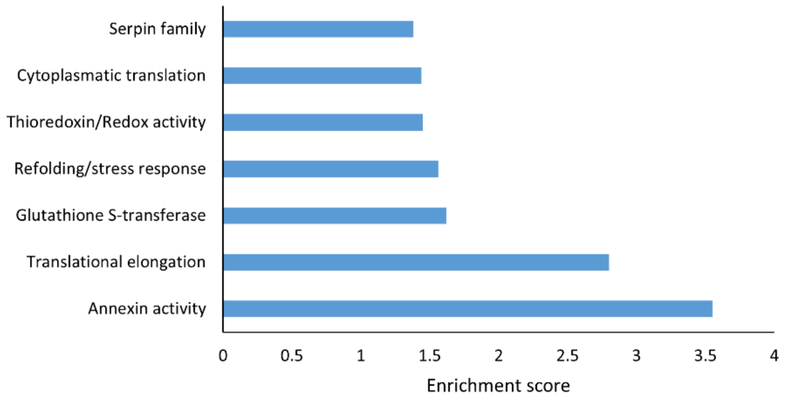

Figure S2. Cluster analysis on proteins differentially expressed between CTRL and infertile patients using DAVID bioinformatics.

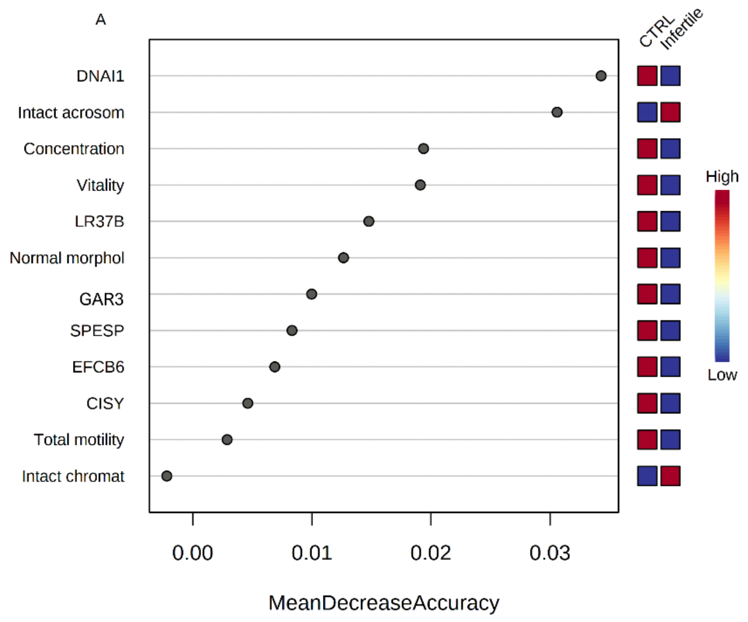

B

The OOB error is 0.215

	CTRL	Infertile	class.error
CTRL	42	8	0.16
Infertile	9	20	0.31

Figure S3. Random forest analysis for sperm functionality parameters (concentration, viability, total motility, normal morphology, chromatin status and acrosome status) and expression of the 6 differentially expressed proteins between CTRL and infertile patients [Citrate synthase (CISY), Dynein axonemal intermediate chain 1 (DNAI1), EF-hand calcium-binding domain-containing protein 6 (EFCB6), Golgi-associated RAB2 interactor protein 3 (GAR3), Leucine-rich repeat-containing protein 37B (LR37B), Sperm equatorial segment protein 1 (SPESP)]. **A** Results expressed in Mean decrease accuracy. **B** Classification table with out-of-bag (OOB) and class errors.

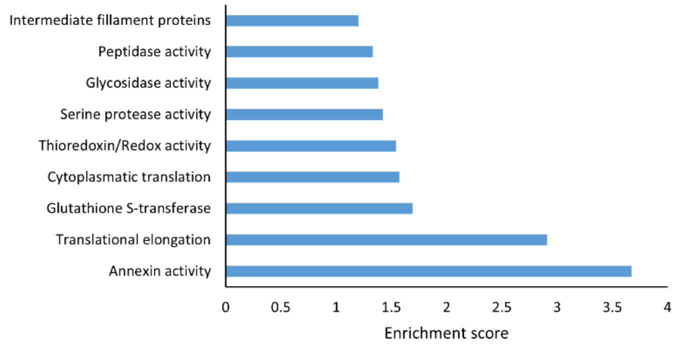

Figure S4. Cluster analysis on proteins differentially expressed between ID and UMI patients using DAVID bioinformatics.

A

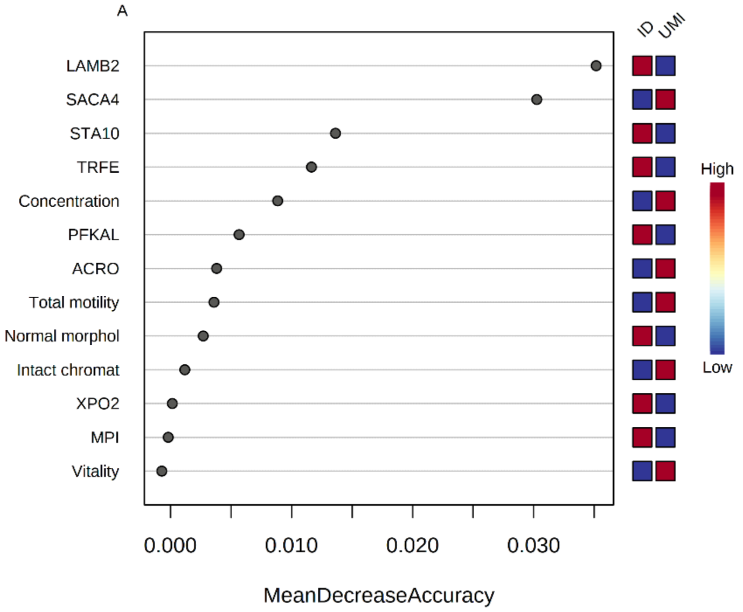

B

The OOB error is 0.276

	ID	UMI	class.error
ID	16	3	0.158
UMI	5	5	0.5

Figure S5. Random forest analysis for sperm functionality parameters (concentration, viability, total motility, normal morphology, chromatin status and acrosome status) and expression of the 6 differentially expressed proteins between ID and UMI patients [Acrosin (ACRO), Sperm acrosome membrane-associated protein 4 (SACA4), Laminin subunit beta-2 (LAMB2), Mannose-6-phosphate isomerase (MPI), ATP-dependent 6-phosphofructokinase liver type (PFKAL), START domain-containing protein 10 (STA10), Serotransferrin (TRFE), Exportin-2 (XPO2)]. **A** Results expressed in Mean decrease accuracy. **B** Classification table with out-of-bag (OOB) and class errors.

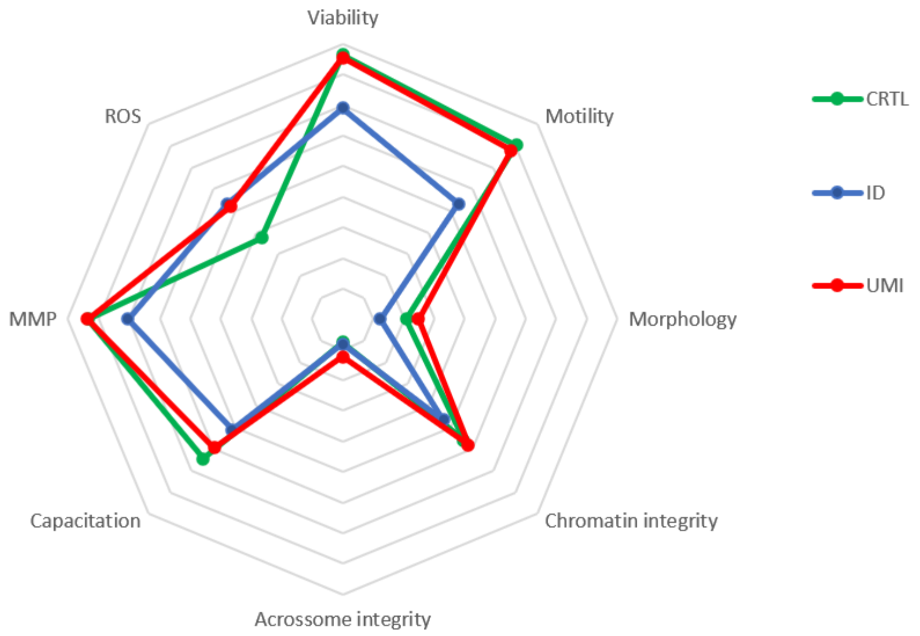

Fig. S6- Sperm functional parameters variation among the three study groups. Results are presented in a radar graph (for each parameter and study group, the results were normalized as follows: mean/highest value × 100). CTRL – healthy controls, ID- idiopathic male infertility, UMI- unexplained male infertility; MMP- Mitochondrial membrane potential; ROS- reactive Oxygen species, specifically mitochondrial superoxide.

Table S1- Study cohort characterization; age, body mass index (BMI) and percentage of individuals performing regular physical exercise, eating healthy, presenting chronic diseases, hypercholesterolemia, COVID-19, allergies, mumps, other diseases, submitted to surgery and with diagnosed depression in the three study groups. All parameters are expressed in percentage, except age and BMI which are presented as mean±SEM. CTRL – healthy controls, ID – idiopathic infertile men, UMI – unexplained infertile men; COVID 19- Corona Virus Disease; *- p≤0.05 in comparison to CTRL; #- p≤0.05 and ###- p≤0.001 in comparison with ID.

	CTRL	ID	UMI	*P value*
Age (n=698)	36.06±0.32	36.69±0.42	36.06±0.62	0.854
BMI (n=694)	26,51±0.20	26.72±0.29	26.75±0.7	0.343
Regular physical exercise (n=694)	222 (55.5%)	93 (48.4%)	41 (40.2%)	0.385
Healthy eating (n=695)	342 (85.3%)	160 (83.3%)	82 (79.4%)	0.461
Chronic diseases (n=688)	52 (13.1%)	33 (17.4%)	9 (8.9%)	0.118
Hypercholesterol (n=678)	124 (31.8%)	81* (42.8%)	37 (37.0%)	0.034
COVID-19 (n=321)	34# (17.4%)	5 (6.3%)	10# (21.3%)	0.032
Allergies (n=695)	103 (25.8%)	41 (21.0%)	12* (11.9%)	0.010
Mumps (n=671)	82 (21.2%)	33 (17.9%)	26 (26.0%)	0.279
Other diseases (n=693)	36 (9.9%)	14 (7.2%)	4 (3.9%)	0.216
Surgery (n=690)	49### (12.3%)	52 (27.4%)	13### (12.7%)	0.000

Table S2- Percentage of patients presenting conditions known to affect fertility in the three study groups. All parameters are expressed in percentage. CTRL – healthy controls, ID – idiopathic infertile men, UMI – unexplained infertile men, STD- sexually transmitted diseases; #- p≤0.05 and ###- p≤0.001 in comparison with ID.

	CTRL	ID	UMI	P value
STD (n=697)	8 (1.9%)	4 (2.0%)	4 (3.9%)	0.494
Urogenital infections and varicocele (n=695)	28### (7.0%)	44 (22.9%)	4### (3.9%)	0.000
Urogenital anomalies (n=694)	3# (0.7%)	6 (3.1%)	0# (0.0%)	0.027
Testicular torsion (n=691)	16 (4.0%)	8 (4.2%)	3 (3.0%)	0.868
Inguinal hernia (n=687)	27 (6.8%)	19 (10.0%)	8 (8.0%)	0.403
Hormonal therapy (n=693)	0 (0.0%)	3 (1.6%)	1 (1.0%)	0.054

Table S3- Exposure to paints, solvents, pesticides, metals, high temperatures, low temperatures, radiation and dust among the three study groups. Results are expressed in percentage of patients exposed in each of the three groups. CTRL – healthy controls, ID- idiopathic male infertility, UMI- unexplained male infertility.*- p≤0.05 in comparison to CTRL; #- p≤0.05 in comparison with ID.

Exposures	CTRL	ID	UMI	*P value*
Paints (n=698)	106 (26.4%)	33* (17.0%)	20* (19.4%)	0.025
Solvents (n=674)	87 (22.5%)	31 (16.4%)	15 (15.3%)	0.112
Pesticides (n=698)	23 (5.7%)	9 (4.6%)	8 (7.8%)	0.544
Metals (n=677)	123# (31.5%)	40 (21.4%)	31# (31.3%)	0.036
High temperature (n=679)	93 (23.7%)	40 (21.3%)	22 (22.2%)	0.796
Low temperature (n=673)	63 (16.2%)	21 (11.3%)	17 (17.2%)	0.242
Radiation (n=677)	14 (3.6%)	9 (4.8%)	5 (5.0%)	0.715
Dust (n=680)	141 (35.8%)	60 (32.3%)	35 (35.0%)	0.705

a

Table S4- Consumption of alcohol and tobacco among the three study groups. Consumption of alcohol is divided in yes (daily consumption), no and occasional (oc.). Tobacco consumption is divided in yes (currently consuming), no and ex-smoker (for at least 3 months). Results are expressed in number of patients in each of the three groups. CTRL – healthy controls, ID- idiopathic male infertility, UMI- unexplained male infertility

Consumption	CTRL			ID			UMI			P value
	yes	no	Oc.	yes	no	Oc.	yes	No	Oc.	
Alcohol (n=697)	55	70	214	30	34	276	16	18	68	0.974
	yes	no	ex-smoker	yes	no	ex-smoker	yes	No	ex-smoker	
Tobacco (n=698)	109	215	78	62	98	34	32	59	21	0.732

Table S5 – Hospital Anxiety and Depression Score (HADS) for anxiety and depression on the three study groups. Results are presented as mean±SEM and percentage of patients with diagnosed depression $$- p≤0.01 in comparison with UMI. HADS scores for both Depression and anxiety symptoms are [0-7 (Normal) 8-10 (Mild) 11-15 (Moderate) 16-21 (Severe)]. CTRL – healthy controls, ID –idiopathic infertile men, UMI –unexplained infertile men.

	CTRL	ID	UMI	P value
Anxiety (HADS Score)	5.36±0.16 (384)	5.59±0.25 (183)	5.34±0.31 (97)	0.897
Depression (HADS Score)	3.03±0.13 (384)	2.83±0.19 (183)	3.30±0.30 (97)	0.393
Diagnosed depression (Survey; n=696)	12[$$] (3.0%)	8[$$] (4.1%)	11 (10.8%)	0.003

Table S6 – Correlation between sperm functional parameters and patients' health among the three study groups (CTRL – healthy controls, ID- idiopathic male infertility, UMI- unexplained male infertility). The colour scale represents the "r" value obtained by the Spearman correlations. The red tones stand for positive correlations while the blue tones stand for negative ones.

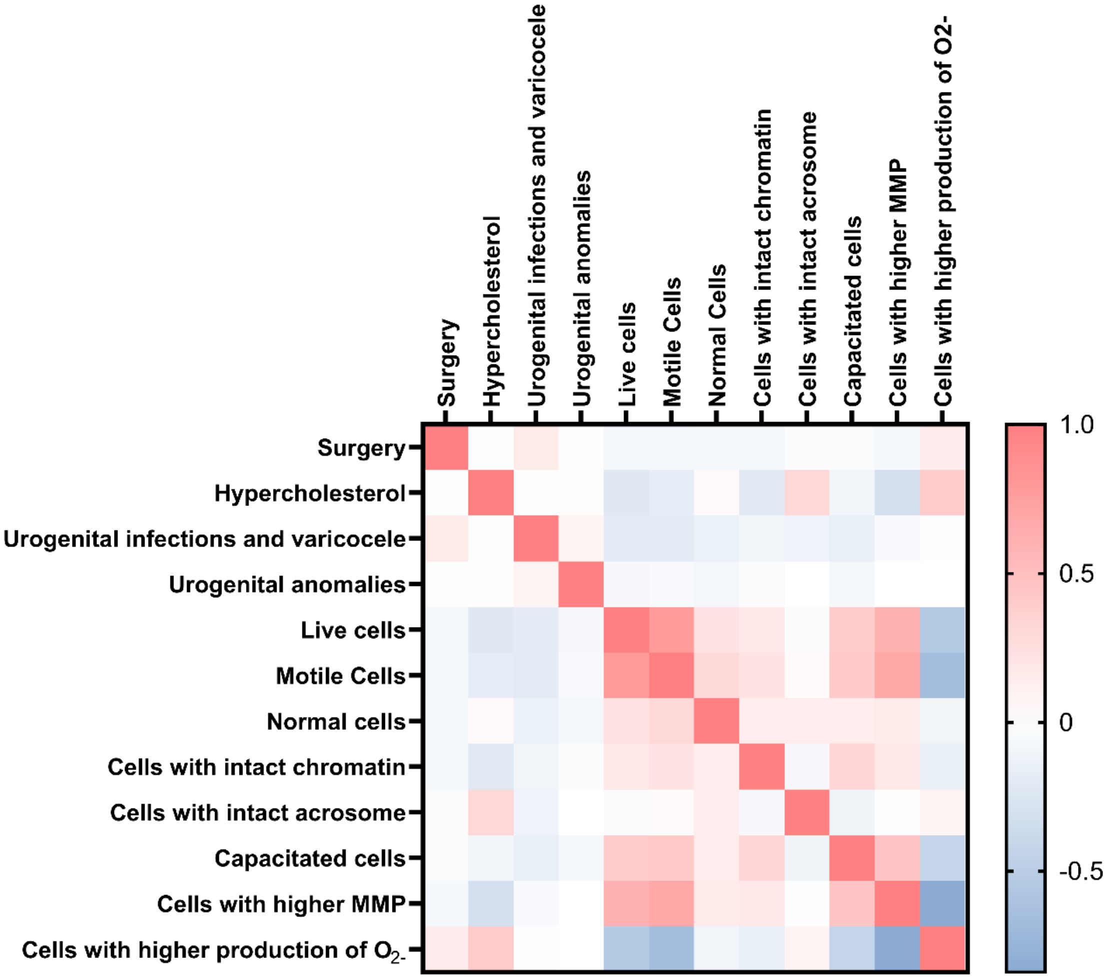

Supplement: Supplementary file 1 [file biomolecules-13-01462-s001.zip › 0929 changed orders by authors/supplementary figures and tables.pdf]
